# An Optimized Human Erythroblast Differentiation System Reveals Cholesterol‐Dependency of Robust Production of Cultured Red Blood Cells Ex Vivo

**DOI:** 10.1002/advs.202303471

**Published:** 2024-03-13

**Authors:** Enyu Wang, Senquan Liu, Xinye Zhang, Qingyou Peng, Huijuan Yu, Lei Gao, An Xie, Ding Ma, Gang Zhao, Linzhao Cheng

**Affiliations:** ^1^ Department of Hematology The First Affiliated Hospital of USTC Division of Life Sciences and Medicine University of Science and Technology of China Hefei Anhui 230001 China; ^2^ Blood and Cell Therapy Institute Anhui Provincial Key Laboratory of Blood Research and Applications University of Science and Technology of China Hefei Anhui 230027 China; ^3^ Department of Electronic Engineering and Information Science University of Science and Technology of China Hefei Anhui 230027 China; ^4^ School of Basic Medical Sciences Division of Life Sciences and Medicine University of Science and Technology of China Hefei Anhui 230027 China; ^5^ Division of Hematology Johns Hopkins University School of Medicine Baltimore MD 21205 USA

**Keywords:** cholesterol, erythroblasts, RBC, terminal maturation

## Abstract

The generation of cultured red blood cells (cRBCs) ex vivo represents a potentially unlimited source for RBC transfusion and other cell therapies. Human cRBCs can be generated from the terminal differentiation of proliferating erythroblasts derived from hematopoietic stem/progenitor cells or erythroid precursors in peripheral blood mononuclear cells. Efficient differentiation and maturation into cRBCs highly depend on replenishing human plasma, which exhibits variable potency across donors or batches and complicates the consistent cRBC production required for clinical translation. Hence, the role of human plasma in erythroblast terminal maturation is investigated and uncovered that 1) a newly developed cell culture basal medium mimicking the metabolic profile of human plasma enhances cell growth and increases cRBC yield upon erythroblast terminal differentiation and 2) LDL‐carried cholesterol, as a substitute for human plasma, is sufficient to support erythroid survival and terminal differentiation ex vivo. Consequently, a chemically‐defined optimized medium (COM) is developed, enabling robust generation of cRBCs from erythroblasts of multiple origins, with improved enucleation efficiency and higher reticulocyte yield, without the need for supplementing human plasma or serum. In addition, the results reveal the crucial role of lipid metabolism during human terminal erythropoiesis.

## Graphical abstract

Generation of cultured red blood cells (cRBCs) ex vivo represents a potentially unlimited resource for RBC transfusion and other cell therapies. However, efficient differentiation highly relies on human plasma/serum, which exhibits variable potency and complicates clinical translation. The crucial role of lipid metabolism during human terminal erythropoiesis is revealed and establish a new chemically‐defined optimized medium (COM) for robust production of cRBCs.

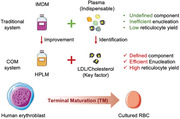

## 1 Introduction

Human red blood cells (RBCs), or erythrocytes, are among the most abundant cells in the body and play a vital role in the transportation of oxygen in the circulatory system.^[^ ^1^ ^]^ Transfusion of donor‐derived RBCs is the most common form of cell therapy in current clinical practice.^[^ ^2^, ^3^ ^]^ Due to limited donor availability, risks of pathogen contamination, and potential allo‐immunization to donor RBCs even after ABO blood‐type matching, ex vivo production of transfusion‐ready cultured RBCs (cRBCs) is a promising replacement for the current fully donor‐dependent therapy.^[^ ^4^, ^5^, ^6^, ^7^ ^]^ The generation of cRBCs ex vivo is a complex process that mainly includes early erythroblast commitment and expansion, followed by terminal erythroid differentiation and reticulocyte maturation.^[^ ^8^ ^]^ In recent decades, considerable effort has been made to generate cRBCs ex vivo from human peripheral blood mononuclear cells (PBMCs),^[^ ^9^, ^10^, ^11^ ^]^ CD34^+^ hematopoietic stem and progenitor cells (HSPCs) ^[^ ^12^, ^13^, ^14^ ^]^ and human pluripotent stem cells (PSCs). ^[^ ^15^, ^16^, ^17^, ^18^, ^19^, ^20^, ^21^, ^22^, ^23^ ^]^ To generate great numbers of cRBCs ex vivo, two objectives must be achieved: 1) extensively expand erythroblasts that can differentiate after massive cell proliferation and 2) efficiently carry out terminal differentiation resulting in large numbers of enucleated erythrocytes.

The first objective, extensively expanding or immortalizing erythroid precursors (erythroblasts), has been largely achieved in recent years. For example, studies have shown that inducible expression of the *HPV16‐E6/E7* oncogene can immortalize human erythroblasts derived from either human PSCs or CD34^+^ cells, generating erythroblast‐like cell lines such as HUDEP2^[^ ^24^ ^]^ and BEL‐A.^[^ ^25^ ^]^ However, after expansion, HPV16‐E6/E7‐transduced erythroblasts carry abnormal karyotypes and only retain the partial capability to differentiate into erythrocytes.^[^ ^26^ ^]^ More critically, significant cell death was observed shortly after the withdrawal of the induced *HPV16‐E6/E7* gene expression to achieve terminal differentiation.^[^ ^27^ ^]^ Alternatively, we have previously described an ex vivo expansion method of human erythroblasts from adult PBMCs through ectopic expression of a single human gene, *BMI1*.^[^ ^10^ ^]^ This approach made it possible to achieve a significant proliferation of human erythroblasts for over one trillion‐fold in 2 months in a defined expansion medium. These extensively expanded erythroblasts can generate enucleated erythrocytes within 8 days upon induction of terminal differentiation using a relatively established culture medium containing human plasma and other more defined factors.^[^ ^5^, ^10^ ^]^

Despite advances in the early stage of cRBC production (i.e., generating great numbers of erythroblasts), the second objective associated with efficient enucleation with a defined culture system remains a major hurdle that needs to be overcome prior to clinical use.^[^ ^5^, ^28^ ^]^ Currently, the most widely used culture condition is Iscove's modified Dulbecco's medium (IMDM) supplemented with human plasma, with or without serum, in addition to more defined factors such as erythropoietin (EPO), holo‐transferrin, insulin, and heparin.^[^ ^29^, ^30^ ^]^ Nonetheless, relatively little progress has been made on how culture media and plasma composition impact terminal differentiation. Of note, human plasma or its derivatives are indispensable to achieving efficient differentiation, as almost all published studies indicate.^[^ ^10^, ^28^, ^29^, ^30^, ^31^, ^32^ ^]^ We reasoned that 1) the basal medium currently used in the terminal maturation stage did not reflect the real metabolites at physiological levels present in circulating human blood, including amino acids, salts, vitamins, glucose, and other small molecules; 2) one or more bioactive macromolecules in human plasma or serum, such as protein complex(es), play an essential role in erythroblast differentiation and reticulocyte maturation. We have long investigated how to identify the key components of human plasma required for erythroblast terminal differentiation to generate enucleated cRBCs ex vivo.

In the present study, we found that a newly developed cell culture system (human plasma‐like medium), mimicking the metabolic profile of human plasma, enhances erythroblast proliferation and increases cRBC yield. Using this advanced human erythroblast differentiation model, we further investigated the role of human plasma in erythroblast terminal maturation and discovered that low‐density lipoprotein (LDL)‐carried cholesterol, as a substitute for human plasma, is sufficient for cRBC production with high efficiency ex vivo. Consequently, we developed a chemically defined optimized medium (COM) enabling robust generation of cRBCs from multiple sources of erythroblasts with improved enucleation efficiency and higher reticulocyte yield compared to culture in traditional media supplemented with human plasma or serum.

## 2 Results

### 2.1 Culture of Erythroblasts in a More Physiologically Relevant Medium Greatly Improves Erythrocyte Maturation and Yield

We hypothesized that multiple bioactive components might be present in human plasma, which are necessary to support erythroblast terminal differentiation and maturation into cRBCs. These components could include proteins (or complexes) that act as ligands or other essential ingredients transported into cells, as well as one or more small molecules present in human plasma that modulate metabolic or other cellular processes. While it is widely recognized that a complex interplay of bioactive factors influences the fate of human cells both in vitro and in vivo, commonly used culture media do not accurately replicate the metabolic composition of human fluids, such as plasma.^[^ ^33^ ^]^ Recently, a new basal medium, human plasma‐like medium (HPLM), was developed to more closely resemble the natural cellular environment by mimicking the metabolite profile of human plasma. HPLM has been shown to better support human cell growth in culture.^[^ ^33^, ^34^, ^35^ ^]^

**Figure 1:**
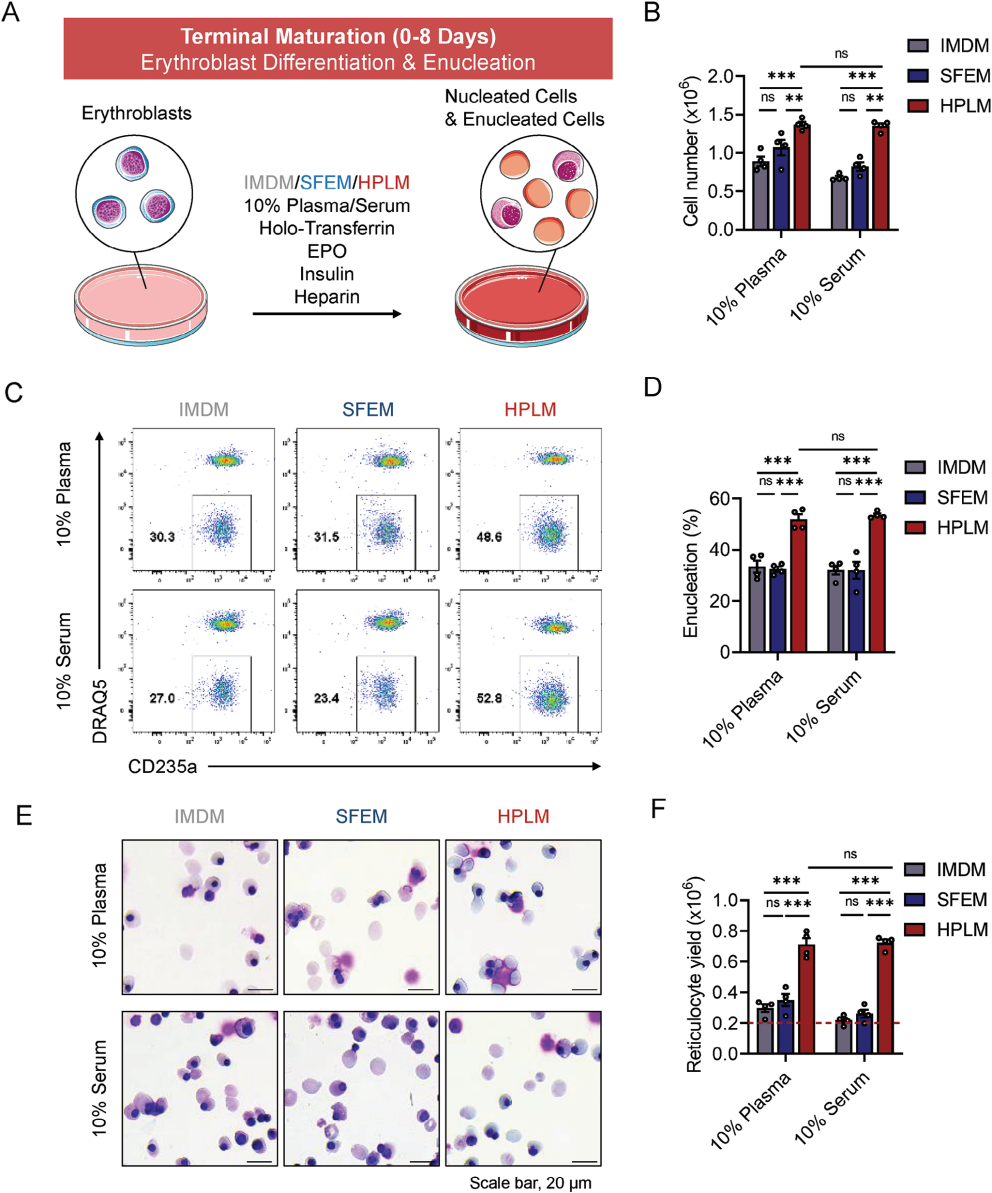
Differentiation of human erythroblasts in a physiological medium greatly improves the erythrocyte yield. A) Schematic diagram of the experimental design. The erythroblasts enriched from human peripheral blood mononuclear cells (PBMCs) were induced to terminal maturation for 8 days in basal medium: Iscove modified Dulbecco medium (IMDM), serum‐free expansion medium (SFEM), or human plasma‐like medium (HPLM), supplemented with 10% human plasma or serum and additional essential components. EPO, erythropoietin. B) The cell number was counted on day 8 via a cell analyzer, and the counting threshold was set for cells larger than 7 µm in diameter (also shown in Figure S2, Supporting Information). C) Flow cytometric analysis of the enucleation rate of differentiated cells on day 8. DRAQ5‐CD235a+ cells indicate enucleated erythrocytes. D) Quantitative analyses of the results shown in C). E) Giemsa staining of differentiated cells. Scale bar, 20 µm. F) Calculation of reticulocyte yield (cell number × enucleation rate). The red dashed line indicates the number of input erythroblasts. The frequency of medium change in the culture medium was every 3 days. Data are shown as the mean ± SEM from four biological replicates. *** *p* < 0.001; **p < 0.01; ns, nonsignificant.

To determine whether HPLM was indeed better at supporting erythroblast terminal differentiation, we compared it with two other basal media. The first was IMDM, which we and others have previously used for erythroid terminal differentiation. ^[^ ^10^, ^32^, ^36^, ^37^ ^]^ The second was serum‐free expansion medium (SFEM), which we used for erythroblast derivation and expansion with appropriate cytokines. We first established human erythroblasts from human PBMCs without CD34^+^ isolation using a previously established platform.^[^ ^10^, ^38^ ^]^ Then, a terminal maturation medium containing three different basal media and common supplements was applied to induce erythroblast differentiation and enucleation, as shown in **Figure** **1**A. First, we examined the differentiation levels throughout the culture period and found that the total cell number reached a plateau at days 6–8, whereas the enucleation rate increased throughout (up to 95%) (Figure S1, Supporting Information). Hence, day 8 was chosen as the time point for measurement and comparison. On day 8 post differentiation, we counted total enucleated reticulocytes and nucleated erythroblasts while excluding smaller pyrenocytes (nuclei encapsulated with membrane, <7 µm in diameter) (Figure S2A,B, Supporting Information). Figure 1B shows that there were more total differentiated cells cultured in HPLM supplemented with either plasma or serum compared to those cultured with IMDM and SFEM basal media. We evaluated the effect of HPLM on enucleation (nucleus removal), the last step of erythroid differentiation, by staining for DRAQ5, which stains DNA in live cells. Figure 1C displays the representative enucleation profiles following the gating strategy (also see Figure S2C, Supporting Information). Quantitative analysis demonstrated that under HPLM conditions, more than 50% of the cells enucleated by day 8 when 10% human plasma or serum was provided.

In contrast, the enucleation rate was approximately 20–30% in the IMDM and SFEM groups (Figure 1D). Cytospin analysis confirmed the improved enucleation (Figure 1E). As expected, the reticulocyte yield was significantly improved in culture with HPLM relative to that in traditional media (Figure 1F). Therefore, we used HPLM basal medium in subsequent studies to investigate the role of other components (supplements or those present in serum) required for erythroblast differentiation and cRBC production ex vivo.

### 2.2 An Optimized Differentiation Protocol Permits cRBC Generation with High Efficiency

**Figure 2:**
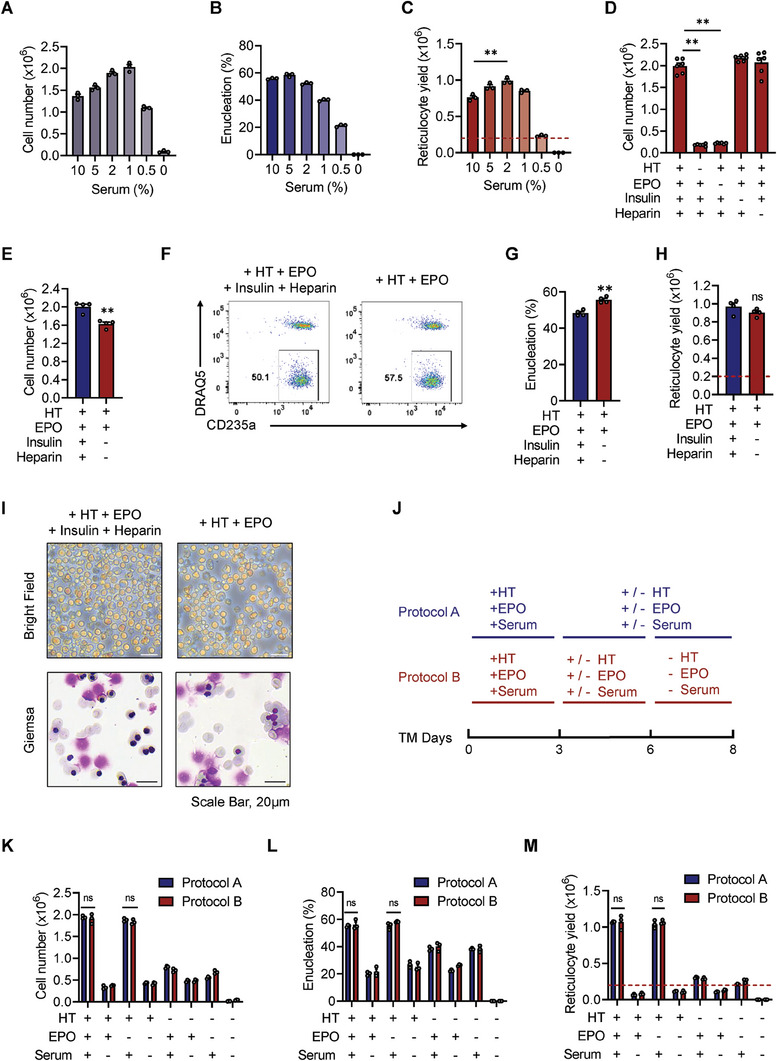
An optimized differentiation protocol permits cRBC generation with high efficiency. A–C) Terminal maturation of PBMC‐derived erythroblasts cultured in diverse concentrations of serum from 10% to 0%. Cell number A), enucleation rate B), and reticulocyte yield C) were assessed. Data are shown as the mean ± SEM from three biological replicates. **p < 0.01. D) The number of differentiated cells after 8 days of terminal maturation in the indicated supplements. Data are shown as the mean ± SEM from six biological replicates. **p < 0.01; ns, nonsignificant. E‐I) Measurement of differentiation efficiency of PBMC‐derived erythroblasts in the absence of insulin and heparin. E) The cell number was counted. F) Flow cytometric analyses of the enucleation rate of PBMC‐derived erythroblasts in different culture media. DRAQ5^‐^CD235a^+^ cells indicate enucleated cells. G) Quantitative analyses of the results shown in F). H) Calculation of reticulocyte yield. The red dashed line indicates the number of input erythroblasts. I) Images of differentiated cells on day 8 in the bright field and Giemsa staining. Scale bar, 20 µm. HT, holo‐transferrin. Data are shown as the mean ± SEM from four biological replicates. **p < 0.01; ns, nonsignificant. J–M) Determination of the time window of serum, EPO and HT supplementation. J) The experimental design. Cell number K), enucleation rate L), and reticulocyte yield M) were estimated. The red dashed lines in the panels indicate the number of input PBMC‐derived erythroblasts. The frequency of medium change in the culture medium was every 3 days. Data are shown as the mean ± SEM from three biological replicates. ns, nonsignificant.

We aimed to determine the optimal concentration of human serum and the roles of other commonly used supplements.^[^ ^10^, ^28^, ^36^, ^37^, ^38^ ^]^ We compared the effects of various serum concentrations (titrated down from 10%) on erythroblast terminal differentiation (**Figure** **2**A–C). We found that a serum concentration of 1% was sufficient for cell growth in HPLM basal medium, but the enucleation rate gradually declined as the serum concentration decreased below 2%. Therefore, we determined that a 2% serum concentration is optimal for cRBC production (Figure 2C), and we used this concentration in subsequent experiments involving serum. We also evaluated the importance of four other components: holo‐transferrin, EPO, insulin, and heparin, which are commonly added to the maturation media. As in previous studies, we found that both high concentrations of holo‐transferrin (500 µg mL^−1^) and EPO are essential for cell proliferation and survival (Figure 2D). In the absence of either insulin or heparin, we observed a slight decrease in total cell number (Figure 2E,I) but a small increase in the enucleation rate (Figure 2F,G), such that the final reticulocyte yield was not significantly different from that in the four‐supplement condition (Figure 2H). We also observed transient proliferation during the first few days after erythroblast differentiation induction, followed by cell growth arrest and terminal enucleation in the next several days. In general, it was optimal on day 8 to harvest all differentiated cells (Figure S3, Supporting Information). Taken together, these findings suggest that a chemically defined HPLM supplemented with 2% human serum, holo‐transferrin, and EPO is sufficient for efficient erythroblast terminal differentiation and enucleation ex vivo.

Next, we attempted to determine the optimal frequency of medium changes during 8 days of terminal differentiation and maturation. We found that refreshing the medium twice (at days 3 and 6) resulted in optimal reticulocyte production on day 8 after terminal maturation induction (Figure S4, Supporting Information). We also investigated when these supplements were critically required during terminal maturation using two different protocols. In the first protocol, all three kinds of components were added at the beginning (day 0), and we omitted one component on day 3 or day 6 when the culture medium was refreshed (Figure 2J). The results showed that human serum and holo‐transferrin should be provided from day 0 to day 6, while EPO was only necessary for the first 3 days without affecting reticulocyte production (Figure 2K–M). We used neonatal leukoreduction filters to remove nuclei and remaining nucleated cells, generating a homogenous population of enucleated cRBCs, as shown in Figure S1C (Supporting Information). Further examination of the concentration of holo‐transferrin revealed that an abundant supply was necessary for the efficient production of reticulocytes (Figure S5, Supporting Information). In summary, we established a highly reproducible and simplified erythrocyte differentiation system that enables the highly efficient production of human cRBCs ex vivo (Figure S6, Supporting Information).

### 2.3 LDL can Essentially Replace Human Plasma/Serum and Ensure Efficient Production of Erythrocytes

**Figure 3:**
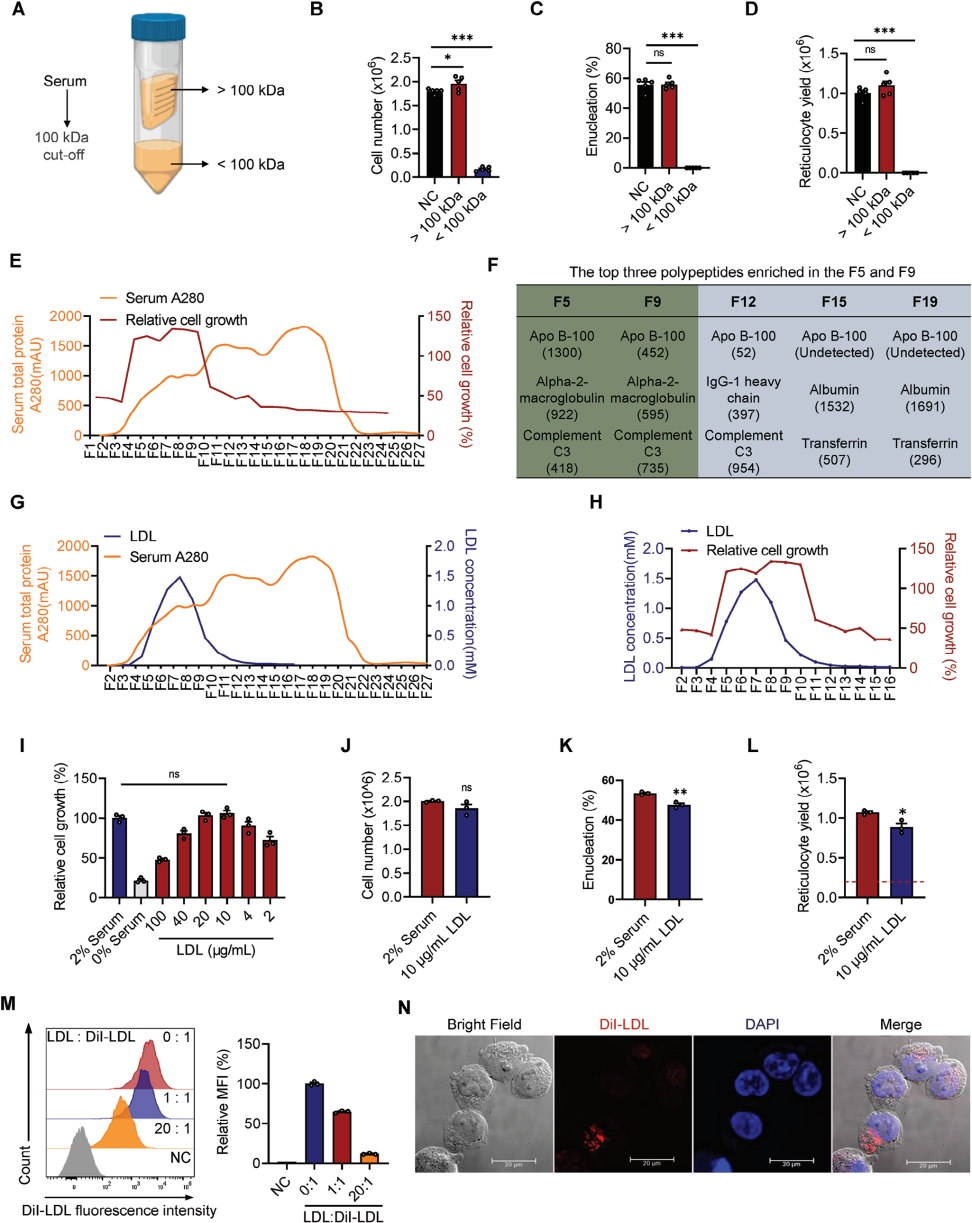
LDL could essentially replace human serum and ensure efficient production of erythrocytes. A–D) Determination of active ingredients in serum that guarantee PBMC‐derived erythroblast survival and terminal maturation. A) Separation of macromolecules and chemicals roughly in human serum by ultrafiltration with a 100 kDa cutoff column. Cell number B), enucleation rate C), and reticulocyte yield D) on day 8 were quantified. Data are shown as the mean ± SEM from three biological replicates. *p < 0.05; ***p < 0.001; ns, nonsignificant. E) The curve of total protein (yellow line) in each fraction separated from human serum by size exclusion chromatography (SEC) and the corresponding growth curve of PBMC‐derived erythroblast treated with different fractions (red line). F) Based on #PSM values, the top three proteins in fractions that support cell survival (F5 and F9, green) were enriched. Among the fractions that cannot support cell survival (F12, F15, and F19, blue), the #PSM of APOB‐100 and the top two ranked proteins in each fraction are also listed. #PSM, the total number of identified peptide sequences (peptide spectrum matches). G) The curve of serum total protein (yellow line) and the corresponding LDL concentration curve (blue line) in each fraction. H) Combination of the growth curve (red line) and LDL concentration curve (blue line). I) Optimization of LDL concentration for terminal maturation. J–L) Comparison of differentiation efficiency between LDL (10 µg mL^−1^) and 2% serum. Cell number J), enucleation rate K), and reticulocyte yield L) on day 8 were quantified. Data are shown as the mean ± SEM from three biological replicates. *p < 0.05; **p < 0.01; ns, nonsignificant. M) Flow cytometric analysis and MFI quantitative analyses of LDL uptake. N) Visualization of LDL uptake (red), and nuclei were counterstained with DAPI (blue). Scale bar, 20 µm.

Even with the HPLM basal medium and essential components such as EPO and holo‐transferrin, human serum (1–2%) is essential for efficient terminal maturation, implying that one or more key components in serum provide essential signaling for cell survival and differentiation. We performed centrifugal filtration using a device with a 100‐kDa molecular weight cutoff to separate small molecules and protein complexes in human serum (**Figure** **3**A). The filtrate (<100 kDa) was unable to support erythroblast survival after differentiation induction, while the concentrates (after dilution to the original volume) retained the full activity of unseparated serum (Figure 3B–D). We used size exclusion chromatography (SEC) to fractionate human serum and assessed the biological effect of each fraction. We developed a simplified assay to identify serum‐containing factors required for cell growth on day 3 after the beginning of terminal differentiation (Figure S7, Supporting Information). In the absence of serum, viable cell numbers gradually decreased over 3 days. Supplementation with 2% serum supported cell growth, which was evident on day 2 and day 3. Using this simplified and high‐throughput assay, which is capable of testing many fractions in triplicate after a 3‐day culture, we found that serum‐derived bioactivity was enriched in fractions F5 to F9 (Figure 3E).

Our preliminary data suggest that the activity resides in one or more protein complexes with molecular weights greater than 100 kDa. We used several different approaches to identify the serum‐containing protein(s). One approach was to identify the polypeptides present specifically or more abundantly in fractions F5 and F9 where the activity resides but with much less presence in fractions F12, F15, and F19 using liquid chromatography‐tandem mass spectrometry (LC‐MS/MS) analysis. Based on the peptide spectrum match (#PSM) values, the top three proteins in fractions that support cell survival (F5 and F9, green) were enriched (Figure 3F and Table S1, Supporting Information). Notably, apolipoprotein B‐100 was the most abundant protein in the bioactive fractions (F5 and F9) but was not or was only slightly present in the negative control groups (F12, F15, and F19). As reported, apolipoprotein B‐100 (molecular weight 500 kDa) is highly enriched in LDL, specifically moving cholesterol around the body to all tissues.^[^ ^39^ ^]^ After directly measuring the total LDL in each fraction, we observed a close correlation between LDL and the serum‐containing activity required for erythroid cell growth (Figure 3G,H). Together, we hypothesize that the cholesterol‐carrying LDL complex or one of its components in the serum is a key factor critical to erythroblast survival and terminal differentiation ex vivo.

We next investigated whether LDL purified from human plasma or serum can replace serum for erythroid commitment and enucleation. First, we determined that an optimal concentration of LDL (10 µg mL^−1^) was sufficient to replace 2% serum and support cell survival and proliferation (Figure 3I). Moreover, LDL had essentially the same capacity as serum to support cell growth during the terminal maturation of erythroblasts, although the enucleation rate and reticulocyte yield were slightly reduced (Figure 3J–L). To confirm that LDL is indeed taken up by erythroblasts for intracellular utilization, we conducted an LDL binding assay. Erythroblasts were coincubated with Dil‐labeled LDL with or without increasing concentrations of unlabeled LDL (Figure 3M). Flow cytometric analysis demonstrated that LDL binds directly to erythroblasts in a dose‐dependent manner (Figure 3M). These data were also corroborated by visualization of the intracellular uptake of LDL after erythroblasts were incubated with Dil‐LDL for 4 h (Figure 3N). The relative intracellular cholesterol level during days 0–3 of terminal maturation with serum or LDL was also higher than that without lipid supplementation (Figure S8, Supporting Information). In conclusion, LDL purified from human plasma can essentially replace 2% unfractionated serum for efficient generation of erythrocytes in culture.

### 2.4 Multiomics Analyses Define the Pivotal Role of Lipid Metabolism in Erythroblast Differentiation

**Figure 4:**
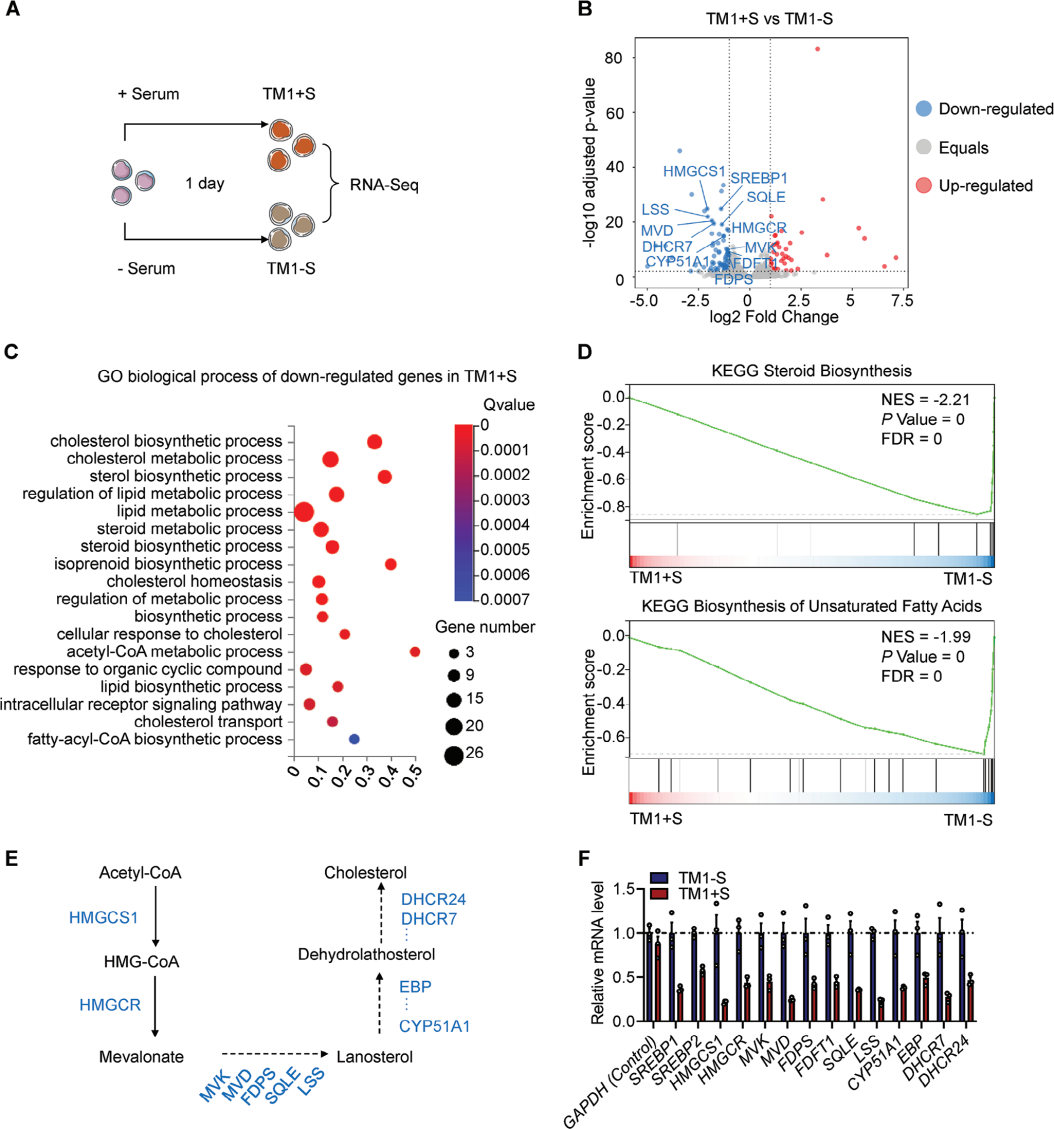
RNA‐sequencing analyses define the pivotal role of lipid metabolism in erythroblast differentiation. A) Schematic diagram of the experimental design for RNA‐seq. PBMC‐derived erythroblasts were differentiated for one day with serum (TM1+S) or without serum (TM1‐S) and collected for transcriptome analysis. B) The volcano plot shows significant differentially expressed genes (|log2FC | ≥ 1 and Q value ≤ 0.01) in cultured erythroblasts with (n=3) versus without (n=3) serum for one day, including 80 downregulated genes highlighted in blue and 39 upregulated genes highlighted in red. Of those downregulated genes, 11 genes related to cholesterol synthesis are labeled. C) GO biological process enrichment analysis was performed on downregulated genes in the TM1+S group (the blue dots in B), revealing a strong correlation with the lipid metabolism process. The bubble plot shows the results of the top 18 terms, with color representing the Q value and size representing the number of genes in the corresponding term. D) GSEA indicates selected gene sets enriched in the TM1+S group compared to the TM1‐S group. Steroid biosynthesis and biosynthesis of unsaturated fatty acids from the KEGG database are shown. E) Schematic representation of the cholesterol biosynthetic pathway. The cholesterol biosynthetic enzymes are highlighted in blue. F) Quantification of the relative mRNA expression levels of the indicated cholesterol biosynthetic genes. Genes were normalized to GAPDH. Data are shown as the mean ± SEM from three biological replicates.

To identify the serum‐derived factor(s) that affect erythroblast differentiation, we performed RNA‐sequencing (RNA‐seq) analysis on cultured erythroblasts with or without 2% serum for one day (**Figure** **4**A; Figure S7, Supporting Information). After 1 day, the cell numbers under the two conditions were similar. This allowed us to capture some of the early changes leading to an understanding of why serum is needed. Compared to control erythroblasts without serum (TM1‐S), we identified a total of 39 upregulated genes and 80 downregulated genes (|log2FC| ≥ 1 and Q value ≤ 0.01) in the TM1+S group with serum 1 day after induction of terminal differentiation (Figure 4B; Figure S9, Supporting Information). Gene Ontology (GO) (Figure 4C) and Kyoto Encyclopedia of Genes and Genomes (KEGG) (Figure 4D) pathway analyses of the differentially expressed genes were conducted. The data revealed that the downregulated genes were strongly enriched in lipid metabolic processes, particularly those associated with the biosynthesis of steroids and cholesterol (Figure 4C). To further investigate the biological significance of lipid metabolism, we conducted gene set enrichment analysis (GSEA) and found a significant downregulation of the biosynthesis of steroids and unsaturated fatty acids in erythroblasts cultured in serum‐containing medium (Figure 4D). We then focused on analyzing the mRNA levels of representative genes involved in the cholesterol synthesis pathway (Figure 4E) and found that their expression levels dramatically decreased in the absence of serum (Figure 4F). Our transcriptomic analysis of differentiating human erythroblasts is consistent with a recent study using a mouse model.^[^ ^40^ ^]^ In this mouse study, the authors found that cholesterol synthesis is downregulated during terminal erythropoiesis and that insufficient or excess cholesterol impairs normal erythropoiesis. Taken together with these findings, we propose that lack of plasma/serum led to stress‐induced upregulation of genes encoding the key enzymes required for endogenous cholesterol biosynthesis; human erythroblasts entering the terminal differentiation stage cannot synthesize cholesterol effectively and highly rely on the uptake of exogenous cholesterol for efficient maturation. This notion is also strengthened by the observation that the amounts of many lipoproteins present in the 2% serum‐containing medium significantly decreased after overnight culture (Figure S10, Supporting Information). Taken together, our transcriptomic and proteomic analyses point to the pivotal role of lipid metabolism in erythroblast survival/proliferation and the high dependency of cRBC production on exogenous cholesterol supplemented by LDL in the cell culture medium.

### 2.5 Cholesterol Dependency of Erythroblast Differentiation was Verified via Disruption of Intercellular Cholesterol Transport and Synthesis

**Figure 5:**
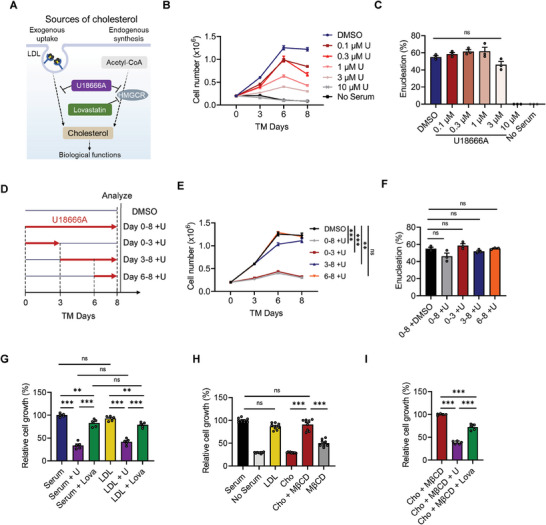
The cholesterol dependency of erythroblast differentiation was verified via disruption of intercellular cholesterol transport and synthesis. A) Schematic diagram showing the two sources of cholesterol, exogenous uptake and endogenous synthesis, and the targets of the inhibitors U18666A and lovastatin. B,C) The effect of the intercellular cholesterol transport inhibitor U18666A on terminal maturation. Cell number B) and enucleation rate C) were quantified. Erythroblasts were derived from PBMC. Data are shown as the mean ± SEM from three biological replicates. D‐F) Terminal maturation of PBMC‐derived erythroblasts after treatment with 3 µM U18666A during different periods. D) Schematic diagram of the experimental design. U18666A was supplemented from day 0–8, day 0–3, day 3–8, or day 6–8. Cell number E) and enucleation rate F) were quantified. Data are shown as the mean ± SEM (n = 3). ***p < 0.001; ns, nonsignificant. G) The effect of U18666A (3 µM) or lovastatin (3 µM) on cell growth in terminal maturation medium supplemented with 2% serum or 10 µg mL^−1^ LDL. Data are shown as the mean ± SEM (n = 5). ***p < 0.001; **p < 0.01; ns, nonsignificant. H) Quantification of relative cell growth in terminal maturation supplemented with 2% serum, 10 µg mL^−1^ LDL, 2.5 µM cholesterol, 2.5 µM cholesterol + 0.1 mg mL^−1^ methyl‐β‐cyclodextrin (MβCD), or 0.1 mg mL^−1^ MβCD. Data are shown as the mean ± SEM (n = 9). ***p < 0.001; ns, nonsignificant. I) The effect of U18666A (3 µM) or lovastatin (3 µM) on cell growth in terminal maturation medium supplemented with 2.5 µM cholesterol + 0.1 mg mL^−1^ MβCD. Data are shown as the mean ± SEM (n = 5). ***p < 0.001. U, U18666A; Lova, lovastatin; Cho, cholesterol; MβCD, methyl‐β‐cyclodextrin.

To investigate the effect of cholesterol levels on erythroblast differentiation, we used the cholesterol transport inhibitor U18666A, which has been shown to block cholesterol transport from lysosomes to the endoplasmic reticulum while leaving exogenous LDL binding and internalization unaffected.^[^ ^41^ ^]^ After detailed tests, we determined that 3 µM was the optimal concentration of U18666A without affecting the enucleation rate (**Figure** **5**A–C). We next treated erythroblasts with U18666A at different time points, as shown in Figure 5D, and found that cell proliferation was significantly reduced after the first 3 days of culture with U18666A (Figure 5E). Surprisingly, all groups exhibited a similar enucleation efficiency when U18666A was administered (Figure 5F). However, inhibiting cholesterol transport on day 6 had minimal effects on erythrocyte production (Figure 5E,F). These findings are consistent with our previous results: plasma/serum is required in the early stage of terminal maturation. In other words, it is essential to add exogenous plasma/serum or LDL/cholesterol for the efficient generation of cRBCs.

To further confirm our hypothesis, we employed lovastatin, an inhibitor of endogenous cholesterol synthesis (Figure 5A). We found that lovastatin when supplemented with human serum or LDL had a slight effect on cell growth, although the small difference is statistically significant (Figure 5G). In comparison with U18666A, an inhibitor of cholesterol transport (Figure 5A), lovastatin exhibited less influence on erythroblast growth (Figure 5G). Given that a previous study showed that cholesterol successfully reversed the repressed mouse erythroid differentiation induced by lipoprotein‐deficient serum,^[^ ^40^ ^]^ it seems that cholesterol carried by LDL supports erythroid survival and terminal differentiation. Afterward, we tested cholesterol for erythroblast differentiation and found that cholesterol can also support human erythroid survival and terminal differentiation efficiently, although methyl‐β‐cyclodextrin (MβCD) had to be added to enable cholesterol solubility (Figure 5H). Additionally, U18666A and lovastatin addition in cholesterol conditions also provided similar results as human serum or LDL (Figure 5I). In conclusion, our findings have uncovered previously unknown mechanisms regarding cholesterol metabolism in human erythroblast terminal maturation. In brief, cholesterol synthesis‐related genes are rapidly downregulated during the transition to the terminal differentiation stage. Thus, exogenous lipids, such as cholesterol native carrier LDL, should be supplied to achieve efficient terminal differentiation and erythrocyte production.

### 2.6 A Chemically Defined Physiological Culture Medium Enables Multiple Sources of Erythroblast Differentiation and Maturation Efficiently

**Figure 6:**
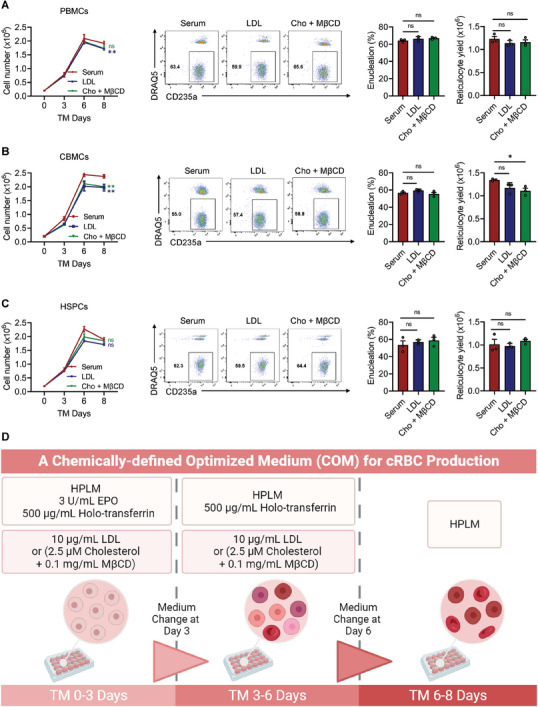
A chemically defined physiological culture medium enables multiple sources of erythroblast differentiation and maturation efficiently. A–C) Terminal differentiation of multiple sources of pro‐erythroblasts in the optimized culture system supplemented with 2% serum, 10 µg mL^−1^ LDL, or 2.5 µM cholesterol + 0.1 mg mL^−1^ MβCD. The sources included PBMCs A), cord blood‐derived mononuclear cells (CBMCs) B), and CD34^+^ hematopoietic stem/progenitor cells (HSPCs) C). DRAQ5^‐^CD235a^+^ cells indicate enucleated cells. Data are shown as the mean ± SEM from three biological replicates. *p < 0.05; ns, nonsignificant. Cho, cholesterol; MβCD, methyl‐β‐cyclodextrin. D) Schematic representation of cRBC production ex vivo in chemically defined optimized medium (COM).

Based on the findings above, we developed a compositionally defined physiological culture medium composed of HPLM, holo‐transferrin, EPO, and LDL or cholesterol, which we refer to as COM. We extended this optimized protocol to human erythroblasts from various sources, including cord blood‐derived mononuclear cells (CBMCs) and CD34^+^ HSPCs. Notably, at least three individuals were included to ensure the reproducibility of our novel approach. As shown in **Figure** **6**A–C, erythroblasts from all these sources, when cultured in COM, effectively enucleated and stably produced reticulocytes with no significant difference compared to the serum‐containing system.

Afterward, we performed further analyses on differentiated cells. Filtration using neonatal leukoreduction filters resulted in 99% removal of nuclei and remaining nucleated cells and yielded a homogenous population of enucleated cRBCs displaying a spheroid morphology that is comparable to native erythrocytes on cytospin (Figure S11A, Supporting Information). All tests on RBC indices, including mean corpuscular volume (MCV), mean corpuscular hemoglobin (MCH), mean corpuscular hemoglobin concentration (MCHC), activity of pyruvate kinase (PK), and ATP, also demonstrated great similarities between cRBCs and native reticulocytes/RBCs^[^ ^6^, ^42^, ^43^, ^44^, ^45^ ^]^ (Figure S11B, Supporting Information). Flow cytometry data showed that cRBCs mainly express adult HBB with different levels of HbF (Figure S11C, Supporting Information). Blood group expression of the cRBCs was assessed by a blood typing test card and compared with the original donor RBCs. Blood group expression was in complete agreement with the original donors (Table S2, Supporting Information). The functional similarities between cRBCs and peripheral blood RBCs indicate that our optimized culture model yields functional enucleated erythroid cells.

In summary, we successfully established an optimized protocol for cRBC production with high enucleation rates, yields, and reproducibility (Figure 6D). Our methods will help researchers understand the regulation of erythroblast differentiation ex vivo and allow for further development of cRBC‐based clinical applications.

## 3 Discussion

There is currently a global imbalance between the supply and demand for red blood cells for transfusion.^[^ ^4^ ^]^ Therefore, there has been a long‐standing effort to produce cultured RBCs with more effective and robust ex vivo methods.^[^ ^46^, ^47^ ^]^ The differentiation of erythroblasts to polychromatic erythroblasts, orthochromatic erythroblasts, and eventually enucleated reticulocytes (cRBCs) is induced by removing stem cell factor (SCF) and glucocorticoid (dexamethasone) while increasing the erythropoietin (EPO) and holo‐transferrin concentration and supplementing with 2–10% human plasma. ^[^ ^10^, ^15^, ^36^, ^48^ ^]^ However, the efficiency of enucleation and erythrocyte yield depends highly on the unpredictable quality of animal‐ or human‐derived components, such as plasma, serum, and albumin.^[^ ^49^ ^]^ In this study, we developed COM, a well‐defined cell culture medium free of plasma, serum, and albumin, which we combined with our optimized protocol for highly efficient production of enucleated cRBCs from multiple sources on a large scale. We believe our novel system will significantly reduce the costs, and increase reproducibility compared with previously published protocols because it eliminates the potential variability associated with plasma. Furthermore, the optimized protocol is likely to be useful for researchers worldwide to explore the molecular mechanisms of erythroid differentiation underlying physiological and pathological conditions.

For reasons that are only partially understood, we and others have observed that human erythroblasts mature in large numbers in vivo when transfused into immunodeficient hosts, but they enucleate with low efficiency when transferred to terminal maturation cultures.^[^ ^6^, ^10^, ^21^, ^50^, ^51^ ^]^ We addressed this challenge by adopting a recently developed physiologically relevant basal medium that mimics the metabolic profile of human plasma, resulting in significantly improved maturation of erythroblasts into cRBCs in the presence of HPLM. Erythroblast proliferation and enucleation are highly dependent on the supplementation of human plasma or serum in maturation cultures.^[^ ^52^, ^53^ ^]^ Although these dependencies were observed long ago, the roles of human plasma or serum in this process are still poorly understood.^[^ ^54^ ^]^ Recent publications have reported that fine‐tuning of cholesterol homeostasis controls mouse erythroid differentiation and that disruption of intercellular cholesterol balance results in defects in mouse terminal maturation in vitro and in vivo.^[^ ^40^ ^]^ In particular, it has been suggested that the presence of dexamethasone predisposes human erythroblasts to impaired lipid metabolism and renders their ex vivo expansion highly dependent on plasma lipoproteins.^[^ ^55^ ^]^ However, the signaling of lipid metabolism that is involved in human erythroblast differentiation ex vivo remains to be identified.^[^ ^56^, ^57^, ^58^, ^59^ ^]^ For the first time, this study showed that the lipid composition of human plasma/serum has a profound effect on human erythroblasts in culture. Furthermore, LDL‐carried cholesterol, as a substitute for human plasma/serum, is sufficient for erythroid survival and differentiation ex vivo. Our data suggest that lipoproteins reduce the susceptibility of pro‐erythroblasts to death induced by growth factor starvation and facilitate their terminal maturation by improving membrane homeostasis.

Although these studies have revealed important discoveries, there are also some limitations. First, we did not examine the roles of individual molecules in HPLM or determine which chemical(s) confer improved cell proliferation and erythrocyte yield. Our results also suggest that the physiological concentration of metabolites may contribute to enhanced differentiation. Second, large‐scale cRBC production in culture dishes is practically impossible, and a bioreactor in larger volume conditions will need to be used in the future to meet clinical requirements. Third, in our novel COM system, while filtered cRBCs displayed a spheroid morphology indicative of a reticulocyte population, the functional characterization between cRBCs and peripheral RBCs, including protein/transcriptome abundance, deformability index, oxygen‐binding capacity, and behaviors in vivo, should be performed in further studies. Last, although physiologic media with metabolite concentrations similar to human plasma are now available, they are not a definitive solution since no model can faithfully capture the full complexity of conditions encountered in the human body. Therefore, 3D^[^ ^12^, ^60^ ^]^ and microfluidic cell cultures,^[^ ^61^, ^62^, ^63^ ^]^ along with improvements in physiologic media, will help to solve the problem of the current culture system.

In summary, we have developed a defined plasma/serum‐free medium and an optimized differentiation procedure for producing human cRBCs ex vivo with a high enucleation rate, yield, and reproducibility. This study reports, for the first time, the essential role of lipid metabolism in erythroblast terminal maturation and demonstrates that LDL‐carried cholesterol confers efficient differentiation. Our findings also provide proof of concept for developing a strategy for GMP‐grade, large‐scale production of cRBCs.

## 4 Experimental Section

### Approval for the Use of Anonymous Donor‐Derived Primary Human Cells

All studies using anonymized primary human cells were conducted in accordance with applicable laws and approved by the Institutional Review Board of the First Affiliated Hospital of the University of Science and Technology of China (approval number 2023KY005).

### Enrichment and Expansion of Primary Erythroblasts

Human PBMCs from adult peripheral blood or CBMCs from cord blood were purified via Ficoll‐Paque PLUS (17144002, Cytiva). CD34^+^ cells were isolated from PBMCs by positive selection using a CD34 MicroBead Kit (130‐046‐702, Miltenyi Biotec) according to the manufacturer's instructions.^[^ ^10^ ^]^ Cells were seeded in a 6‐well plate (4 × 10^6^ cells mL^−1^/well) and cultured in the expansion medium, which mainly consisted of StemSpan SFEM (09650, STEMCELL Technologies) supplemented with GlutaMAXTM (35050079, Gibco), human interleukin‐3 (10 ng mL^−1^; 8194.1, STEMCELL Technologies), human recombinant stem cell factor (SCF; 100 ng mL^−1^; 78062.2, STEMCELL Technologies), dexamethasone (DEX; 1 mmol L^−1^; D2915, Sigma‐Aldrich), and erythropoietin (EPO; 3 U mL^−1^; 287‐TC, R&D Systems). When PBMCs or CBMCs were cultured, the culture medium was changed every 3 days, and ≈day 6, the cell concentration was adjusted to 5 × 10^5^ cells mL^−1^, and the cells were collected around day 8. When CD34^+^ cells were cultured, the cells were collected ≈day 14. The collected cells were further used in subsequent terminal maturation experiments.

### Terminal Maturation of Erythroblasts

As previously described,^[^ ^10^ ^]^ the initial terminal maturation culture system consisted of Iscove's modified Dulbecco's medium (IMDM, Gibco, #21056023) supplemented with 10% human plasma (IPLAWBK2E50ML, Innovative Research), 500 µg mL^−1^ human holo‐transferrin (2914‐HT, R&D Systems), insulin (10 µg mL^−1^; I9278, Sigma‐Aldrich), heparin (4 U mL^−1^; H3149, Sigma‐Aldrich) and EPO (3 U mL^−1^). The advanced terminal maturation culture system was composed of human plasma‐like medium (HPLM; A4899101, Gibco) with human serum (100‐512, GeminiBio) or human low‐density lipoprotein (LDL; 20613ES05, YEASEN) or cholesterol (HY‐N0322, MCE) with methyl‐β‐cyclodextrin (MβCD, HY‐101461, MCE), supplemented with human holo‐transferrin (500 µg mL^−1^) and EPO (3 U mL^−1^) (also shown in Figure S6, Supporting Information and Figure 6D).

### Flow Cytometric Analysis

For enucleation rate analysis, approximately 0.2 × 10^6^ cells were taken from the culture, washed, and resuspended in 20 µL of PBS containing 1% fetal bovine serum. The cells were then stained with primary antibodies and dyes for 30 min at 4 °C in the dark. The primary dyes and antibodies used in this study were Fixable Viability Dye eFluor 450 (65‐0863‐14, Invitrogen), DRAQ5 (424101, Biolegend), and CD235a‐PE (349106, Biolegend). For intracellular hemoglobin analysis, approximately 1 × 10^6^ cells were collected, washed, and fixed in PBS with 0.05% glutaraldehyde for 10 min at room temperature. Then, the cells were washed three times by centrifugation at 1000 × *g* for 5 min with PBS. After that, the supernatants were removed, the cell pellet was resuspended in 0.1% Triton X‐100, and the cells were incubated for 3 min at room temperature. The cells were washed twice and stained with primary antibodies and dyes for 1 h at room temperature. The primary dyes and antibodies used were DRAQ5 (424101, BioLegend), HBB‐PE (sc‐21757PE, Santa Cruz), and HbF‐FITC (MHFH01, Invitrogen). Stained cells were analyzed on a BD LSRFortessa flow cytometer (BD Biosciences), and the data were analyzed with FlowJo v.1.6.2 (FlowJo, LLC).

### Cytospin Preparation

A total of 1.5 × 10^5^ cells in 150 µL of PBS were prepared for cytospin on glass slides (BC014, Biosharp) and spun at 500 × *g* for 1 min using a Cellspin I Centrifuge (Tharmac). The cells were then stained with Wright‐Giemsa solution (Hema 3, 123869, Fisher Scientific) according to the manufacturer's instructions and imaged using a Leica DMi8 inverted microscope.

### Size‐Exclusion Chromatography and Mass Spectrometry

Human serum was centrifuged at 10000 × g for 5 min to prepare and separate it into different fractions by size‐exclusion chromatography on an ÄKTA pure 25 L chromatography system (Cytiva) using eluent in PBS for further cell proliferation tests and mass spectrometry. A Superdex 200 Increase 10/300 GL column (28990944, Cytiva) was used. Selected fractions separated by size‐exclusion chromatography were subjected to mass spectrometry in a Q Exactive Plus mass spectrometer (Thermo Scientific), and the data were processed and quantified using Proteome Discoverer software v2.2 (Thermo Scientific).

### Cell Proliferation Assay

Cell growth was assessed using the Cell Counting Kit‐8 (C0005, TargetMol). Specifically, cells were seeded at an initial density of 5 × 10^4^ 100 µL^−1^/well in 96‐well plates and incubated with the corresponding supplements for 72 h. Next, 10 µL of CCK‐8 was added to each well, and the plate was incubated at 37 °C for 2 h. Absorbance was detected at 450 nm using a SpectraMax Id5 Microplate Reader (Molecular Devices).

### RNA Extraction and Sequencing

Erythroblast cells cultured in terminal maturation medium with or without serum were sampled at TM1. Total RNA was isolated using TRIzol (15596026, Invitrogen), followed by quantification and quality control using a NanoPhotometer NP80 (IMPLEN). RNA samples with RNA Integrity Number (RIN) > 10 were used to construct cDNA libraries with the MGIEasy RNA Library Prep Kit (1000006384, MGI). Paired‐end 150 bp reads were generated and sequenced on the DNBSEQ platform (BGI‐Shenzhen).

### Gene Expression Analysis

The sequencing data were filtered with SOAPnuke (v1.5.2) by i) removing reads containing sequencing adapters; ii) removing reads whose low‐quality base ratio (base quality less than or equal to 5) was more than 20%; and (iii) removing reads whose unknown base (“N” base) ratio was more than 5%. Afterward, clean reads were obtained and stored in FASTQ format. The clean reads were mapped to the human reference genome GRCh38.p13 using Bowtie2 (v2.2.5), and then the expression level of the gene was calculated by RSEM (v1.2.8) with default settings. Essentially, differentially expressed genes were identified by DESeq2 (v1.4.5) with a threshold of adjusted *p* value ≤0.01 and absolute value of log2‐fold change >1. The results were visualized by a volcano plot using ggplot2 (v3.3.6) and ggrepel (v0.9.2).

### Gene Annotation and Enrichment Analysis

To investigate the potential enrichment of differentially expressed genes in specific metabolic pathways, molecular functions, or biological processes, Gene Ontology (GO) (http://www.geneontology.org/) and Kyoto Encyclopedia of Genes and Genomes (KEGG) (https://www.kegg.jp/) enrichment analyses were conducted using the Phyper tool (https://en.wikipedia.org/wiki/Hypergeometric_distribution) based on the hypergeometric test. The significance levels of enriched terms and pathways were corrected by the Bonferroni method with a stringent threshold (Q value ≤0.05). Additionally, each KEGG pathway or GO term was defined as a gene set, and gene set enrichment analysis (GSEA) was implemented using the Java application available from The Broad Institute (www.broadinstitute.org/gsea/). Gene sets with FDR values <0.05 were considered statistically significant.

### LDL Uptake Assay

To measure LDL binding and uptake by erythroblasts, cells were incubated in HPLM with 10 µg mL^−1^ DiI‐LDL (20614ES76, YEASEN) and 0, 10, or 200 µg mL^−1^ LDL for 4 h at 37 °C in the dark. After incubation, the cells were collected and washed three times with PBS. Mean fluorescence intensity (MFI) was analyzed by flow cytometry. For cellular fluorescence imaging of LDL uptake, cells were fixed with 4% paraformaldehyde, stained with DAPI for 15 min, and then imaged using a Leica DMi8 inverted microscope.

### Statistical Analysis

Data are presented as the mean ± standard error of the mean (SEM). All statistical analyses were performed using GraphPad Prism 8. An unpaired *t* test with Welch's correction was used for the analysis of two groups, while Brown–Forsythe and Welch ANOVA tests with multiple comparisons were used for the analysis of three or more groups. Experiments were replicated at least three times. Values of *p* < 0.05 were considered statistically significant (*p < 0.05; **p < 0.01; ***p < 0.001); p > 0.05 indicated nonsignificant results (ns).

## Conflict of Interest

S. Liu and L. Cheng are inventors on a patent application submitted by the University of Science and Technology of China. All other authors declare that they have no competing interests.

## Supporting information

Supporting Information

## Data Availability

The data that support the findings of this study are available from the corresponding author upon reasonable request.
