# Supplementary material for: An Optimized Human Erythroblast Differentiation System Reveals Cholesterol‐Dependency of Robust Production of Cultured Red Blood Cells Ex Vivo
Source: Adv Sci (Weinh). 2024 Mar 13;11(22):2303471. doi: 10.1002/advs.202303471 (PMC11165465; doi:10.1002/advs.202303471)
Supplement: Supplementary file 1 — Supporting Information [file ADVS-11-2303471-s001.pdf]

ADVANCED SCIENCE Open Access

Supporting Information

for *Adv. Sci.*, DOI 10.1002/advs.202303471

An Optimized Human Erythroblast Differentiation System Reveals Cholesterol-Dependency of Robust Production of Cultured Red Blood Cells Ex Vivo

*Enyu Wang, Senquan Liu**, Xinye Zhang, Qingyou Peng, Huijuan Yu, Lei Gao, An Xie, Ding Ma, Gang Zhao and Linzhao Cheng**

Supporting Information

An optimized human erythroblast differentiation system reveals cholesterol-dependency of robust production of cultured red blood cells *ex vivo*

Enyu Wang[#], Senquan Liu[#,], Xinye Zhang, Qingyou Peng, Huijuan Yu, Lei Gao, An Xie, Ding Ma, Gang Zhao, and Linzhao Cheng**

E. Wang, S. Liu, D. Ma, L. Cheng
Department of Hematology, The First Affiliated Hospital of USTC, Division of Life Sciences and Medicine, University of Science and Technology of China, Hefei, Anhui, 230001, China

E. Wang, S. Liu, L. Gao, A. Xie, D. Ma, G. Zhao, L. Cheng
Blood and Cell Therapy Institute, Anhui Provincial Key Laboratory of Blood Research and Applications, University of Science and Technology of China, Hefei, Anhui, 230027, China

E. Wang, G. Zhao
Department of Electronic Engineering and Information Science, University of Science and Technology of China, Hefei, Anhui, 230027, China.

S. Liu, X. Zhang, Q. Peng, H. Yu, L. Gao, L. Cheng
School of Basic Medical Sciences, Division of Life Sciences and Medicine, University of Science and Technology of China, Hefei, Anhui, 230027, China

L. Cheng
Division of Hematology, Johns Hopkins University School of Medicine, Baltimore, MD, 21205, USA

*Correspondence
E-mail: liusenquan1988@ustc.edu.cn; lzcheng@ustc.edu.cn
[#]These authors contributed equally

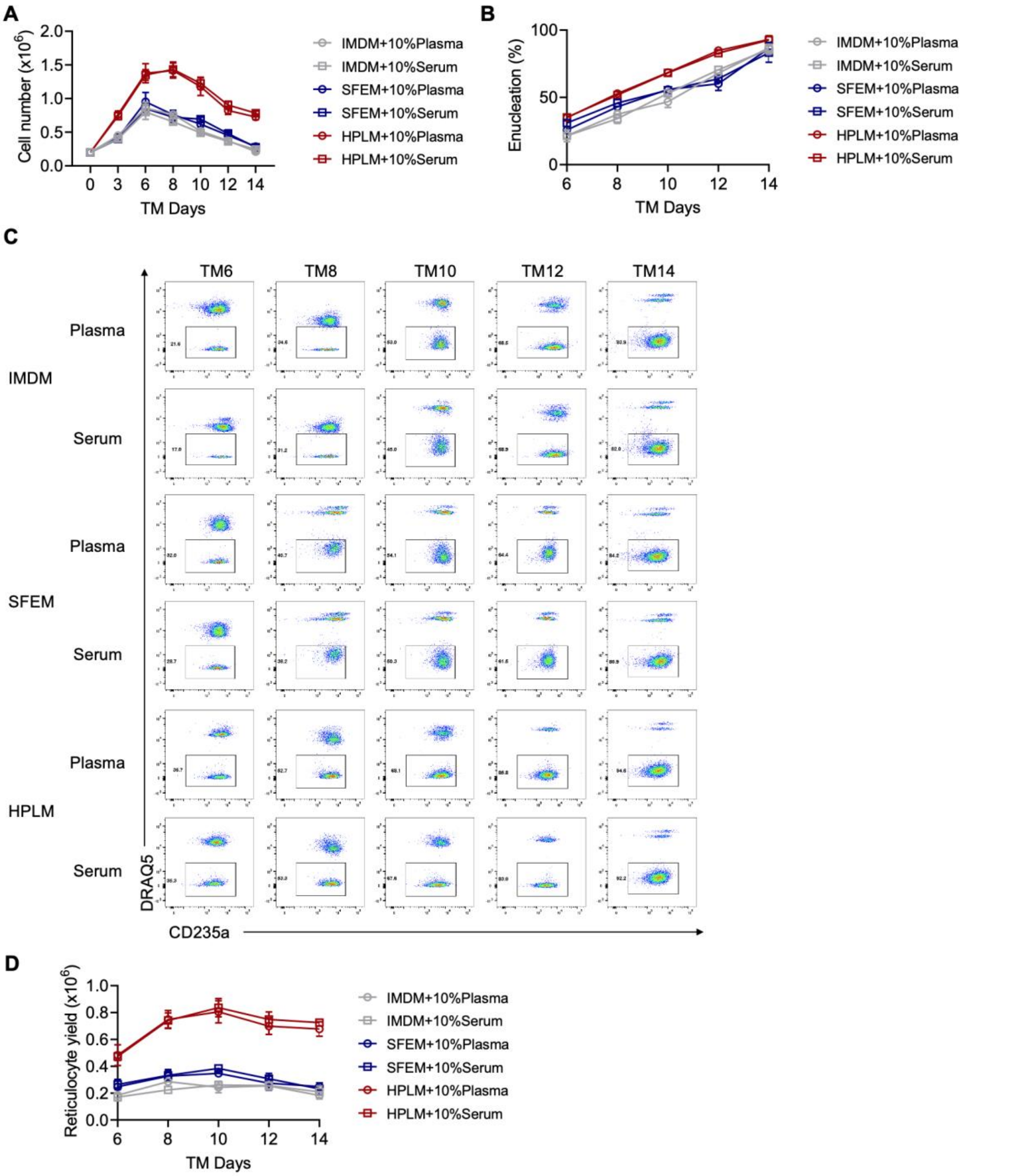

Figure S1. The differentiation level of PBMC-derived erythroblasts in different culture systems. A) Changes in cell number during 14 days of terminal maturation. B) Enucleation rate on days 6, 8, 10, 12, and 14 of terminal maturation. C) Flow cytometric analysis of the enucleation rate of differentiated cells at days 6, 8, 10, 12, and 14. $DRAQ5^{-}CD235a^{+}$ cells indicate enucleated erythrocytes. D) Calculation of reticulocytes. The frequency of medium change in the culture medium was every 3 days.

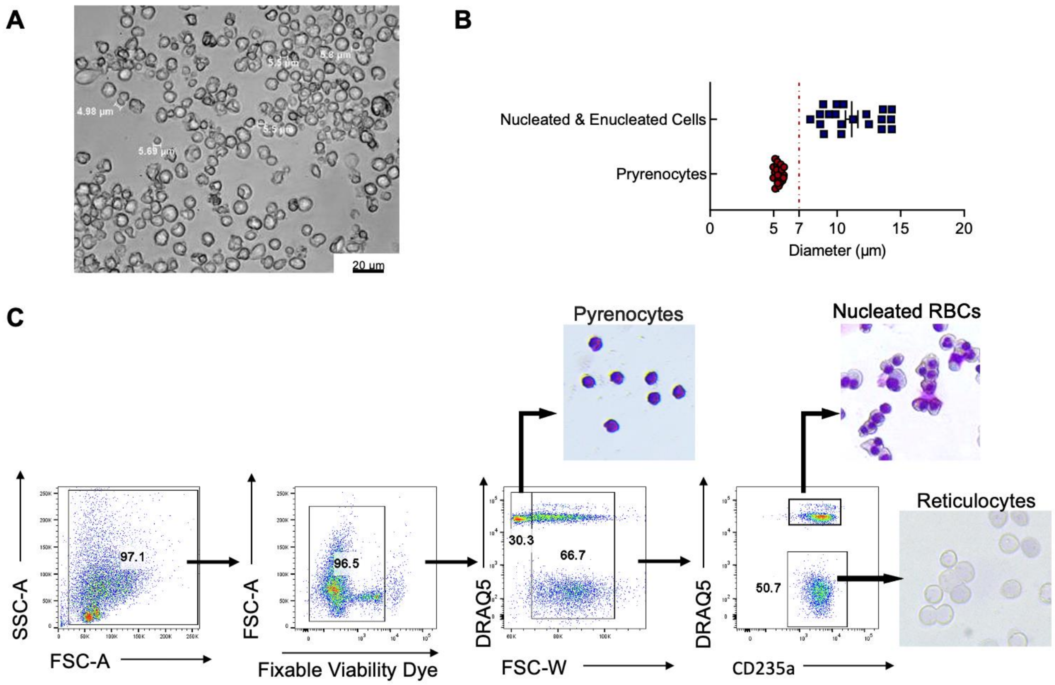

Figure S2. The cell counting and gating strategies for analyses of the differentiated erythroblasts derived from PBMCs. A) Representative images of differentiated cells 8 days after terminal maturation induction. Scale bar, 20 µm. B) Scatter plot of the cell size distribution of differentiated cells. The red dotted line represents the threshold for cell counting. C) Gating strategies and Giemsa staining for differentiated cells on day 8 of terminal maturation.

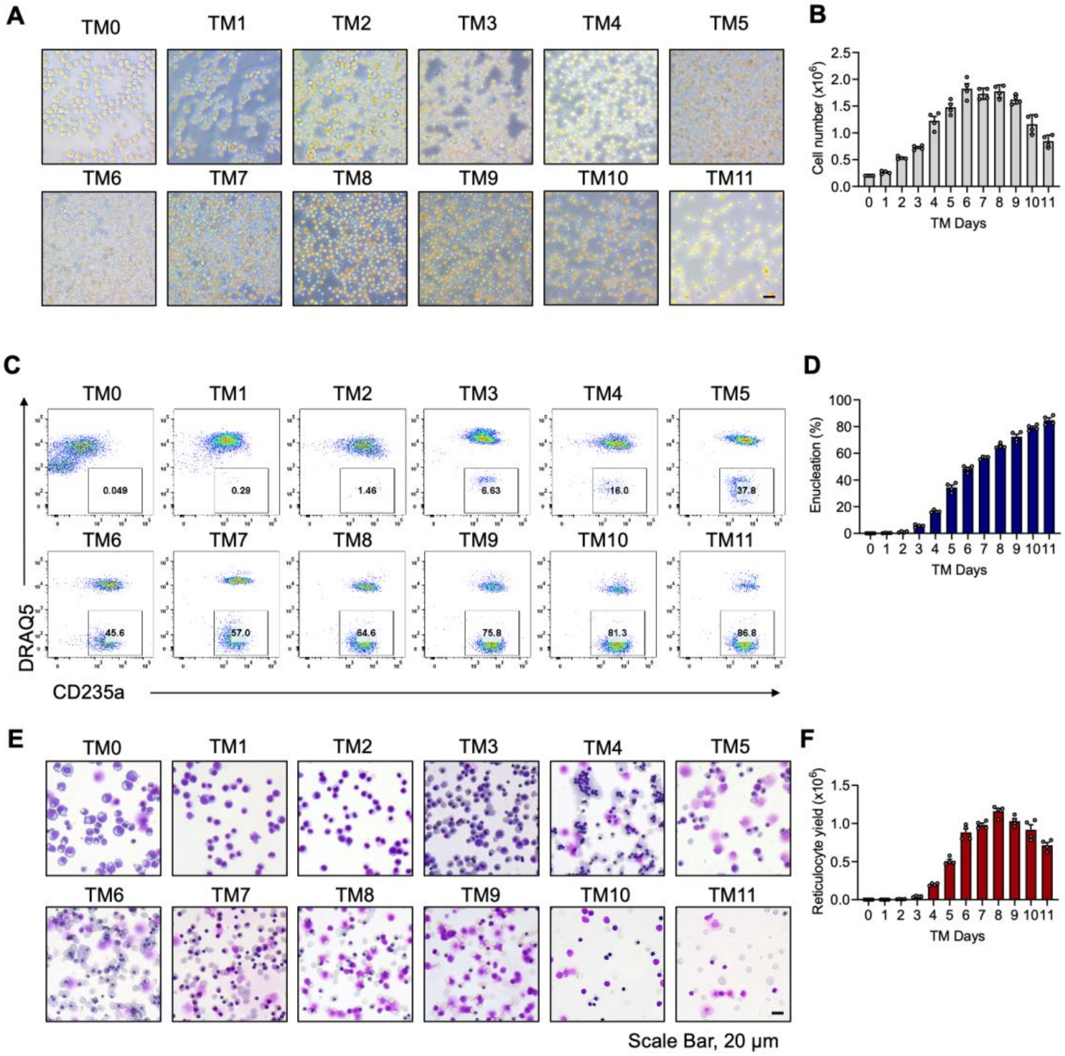

Figure S3. Profiling of terminal maturation indicates that it is optimal on day 8 to harvest differentiated cells from human PBMC-derived erythroblasts under the new culture system. A) Representative bright field images of terminal maturation from day 0 to day 11. Scale bar, 20 μm. B) Cell numbers were counted every day (≥7 μm). C) Flow cytometric analyses of differentiated cells. $DRAQ5^-CD235a^+$ cells indicate enucleated cells. D) Statistical analyses of flow cytometry results. E) Representative Giemsa staining of differentiated cells during terminal maturation. Scale bar, 20 μm. F) The daily production of reticulocytes during the terminal maturation of erythroblasts.

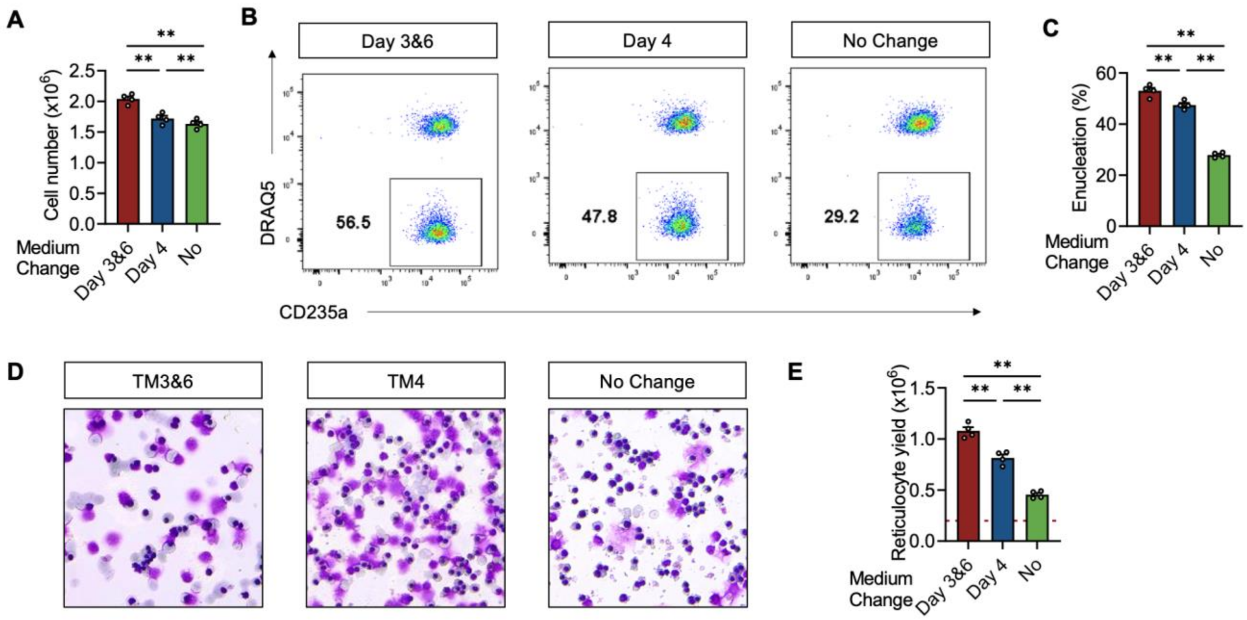

Figure S4. Determination of the frequency of medium changes for efficient production of reticulocytes. A) Total cell number was counted on day 8. The media were changed on day 3 & 6, or day 4, or without change. B) Flow cytometric analyses of enucleation rates on day 8. C) Quantitative analyses of the results shown in B). D) Giemsa staining of differentiated cells. Scale bar, 20 μm. E) Calculation of reticulocyte yield (cell number × enucleation rate). Erythroblasts were derived from PBMC. The red dashed line indicates the number of input erythroblasts. Data are shown as the mean ± SEM from four biological replicates. **, $p < 0.01$.

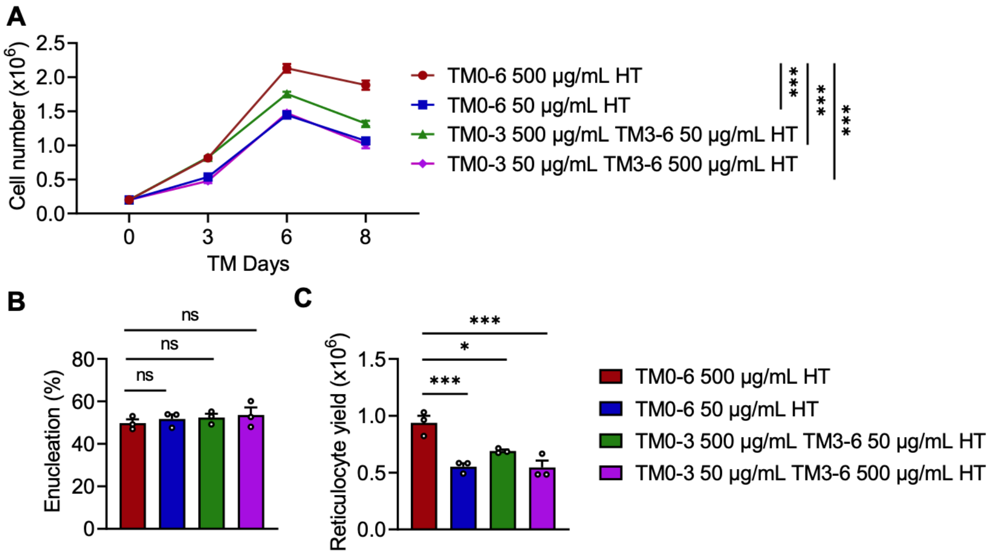

Figure S5. Effects of holo-transferrin concentration and supplementation period on reticulocyte production. A) Cell growth was measured at days 0, 3, 6, and 8. B) Enucleation rates were measured on day 8. C) Reticulocyte yield was calculated as cell number multiplied by enucleation rate. HT, holo-transferrin. Erythroblasts were derived from PBMC. Data are shown as the mean ± SEM from three biological replicates. ***, $p < 0.001$; *, $p < 0.05$; ns, nonsignificant.

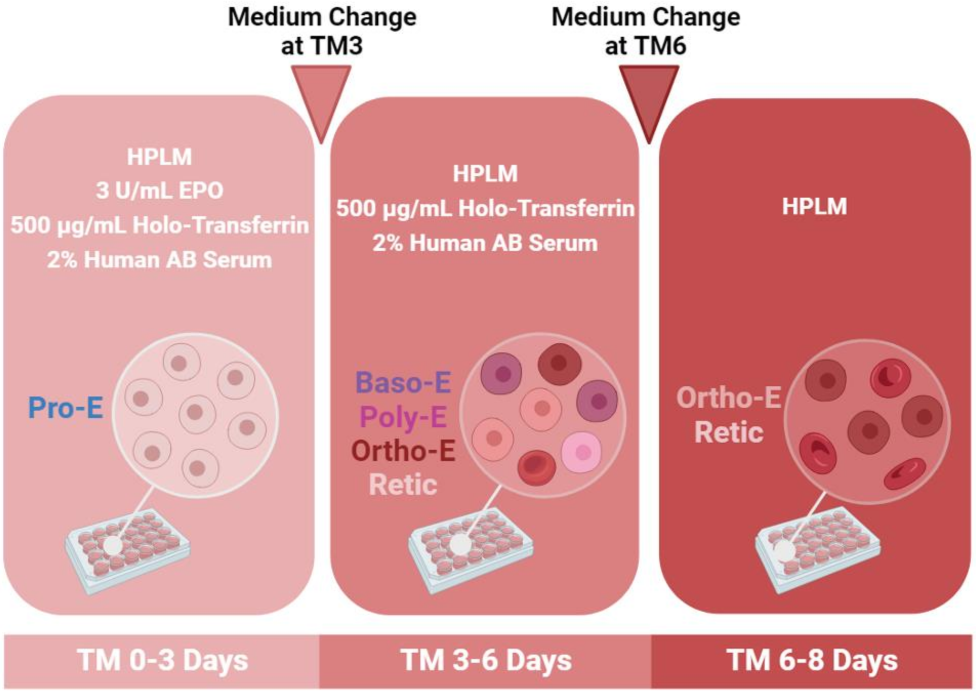

Figure S6. Establishment of a highly optimized, physiologically relevant cell differentiation system containing human AB serum. Erythroblasts were derived from PBMC. Pro-E, proerythroblast; Baso-E, basoerythroblast; Poly-E, polyerythroblast; Ortho-E, orthoerythroblast; Retic, reticulocyte. TM, terminal maturation.

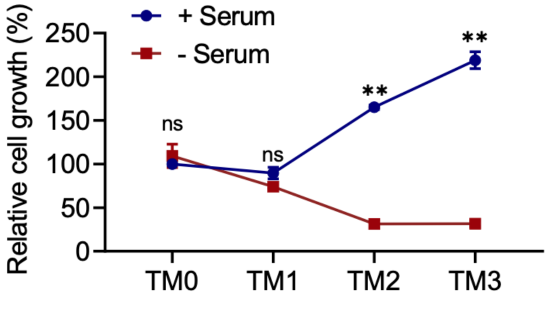

Figure S7. Relative cell growth during days 0-3 of terminal maturation with or without serum. Erythroblasts were derived from PBMC. Data are shown as the mean ± SEM from three biological replicates. **, $p < 0.01$; ns, nonsignificant.

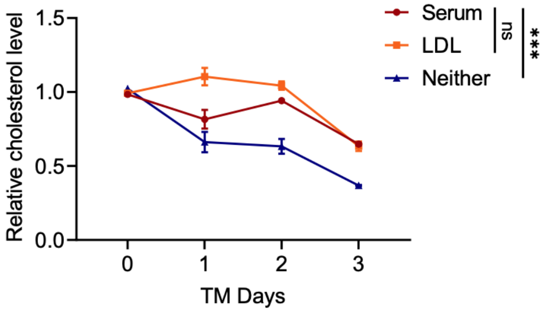

Figure S8. The relative intracellular cholesterol level during days 0-3 of terminal maturation with serum, LDL, or neither. Erythroblasts were derived from PBMC. Data are shown as the mean ± SEM from three biological replicates. ***, $p < 0.001$; ns, nonsignificant.

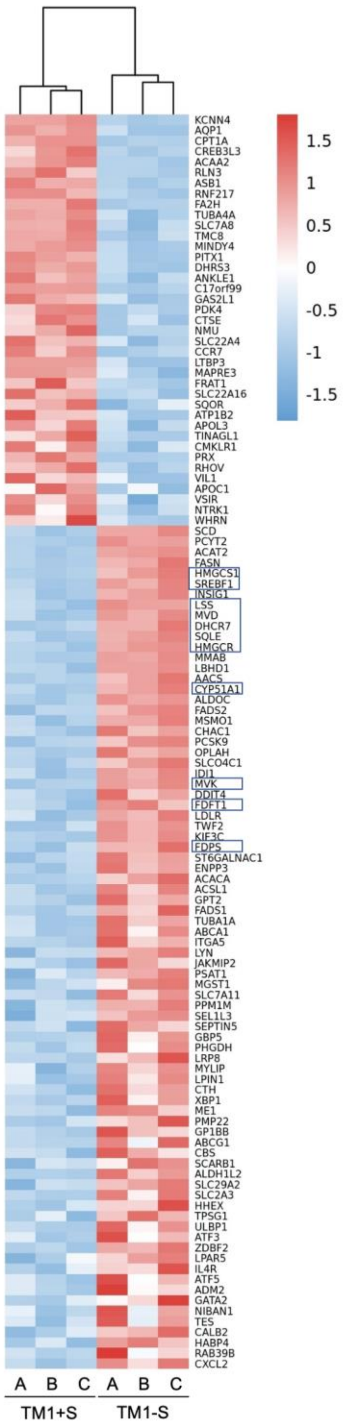

Figure S9. The differentially expressed genes in PBMC-derived erythroblasts induced with or without serum for 1 day. The heatmap shows significant differentially expressed genes (|log2FC| ≥ 1 and Q value ≤ 0.01) in cultured erythroblasts with (n=3) versus without (n=3) serum for one day. Genes related to cholesterol synthesis are labeled in blue squares.

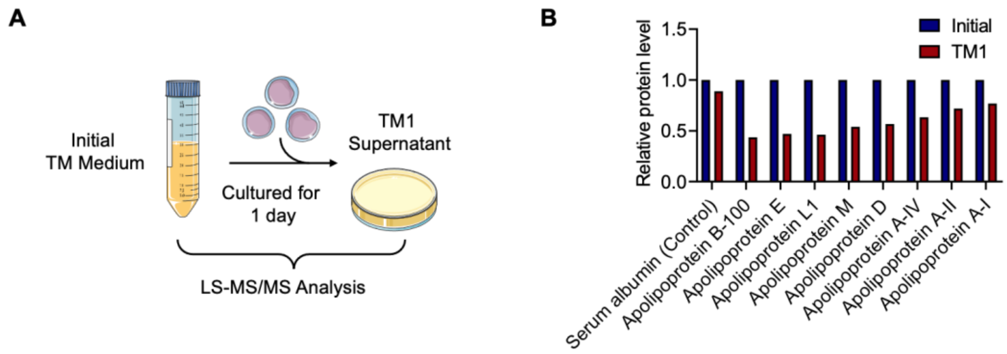

Figure S10. Proteomic analysis of the supernatant from cell culture compared with the initial medium. A) Schematic diagram of the experimental design. B) Comparison of lipoprotein abundance. Erythroblasts were derived from PBMCs. The abundance of lipoproteins in the culture medium was compared using serum albumin as an internal reference control. Initial, the initial terminal maturation medium; TM1, the supernatant from cell culture on day 1 after terminal maturation induction.

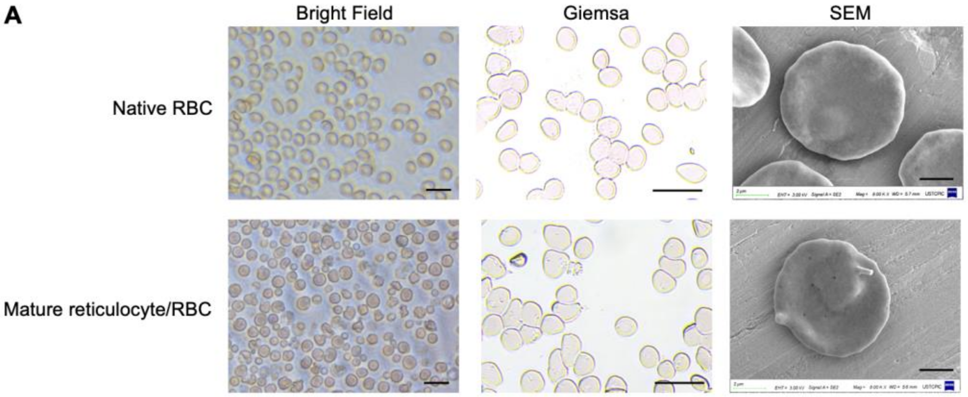

B

Cell type	MCV (fL)	MCH (pg)	MCHC (g/L)	PK (U/gHb)	ATP (μmol/gHb)	References
cRBC (Serum) (n=3)	133.2±2.6	55.8±2.7	422.5±18.2	28.5±3.7	6.2±1.4	Present study
cRBC (LDL) (n=3)	136.7±3.5	57.8±3.0	430.1±21.6	31.8±3.2	6.6±1.7	Present study
cRBC (Cholesterol) (n=3)	135.8±3.8	56.2±2.5	426.5±17.7	25.8±3.7	6.1±1.8	Present study
Native RBC (n=3)	95.5±9.7	30.0±2.2	337.3±21.3	13.0±0.9	5.5±0.4	Present study
cRBC in other Labs	138 ± 4.2	35 ± 1.6	250±5	86 ± 6	—	6
Native Reticulocyte	120-140	—	—	54.2	—	42,43
Native RBC	80-100	26.5-33.5	300-360	11.4±2.8	3.7±0.4	43,44,45

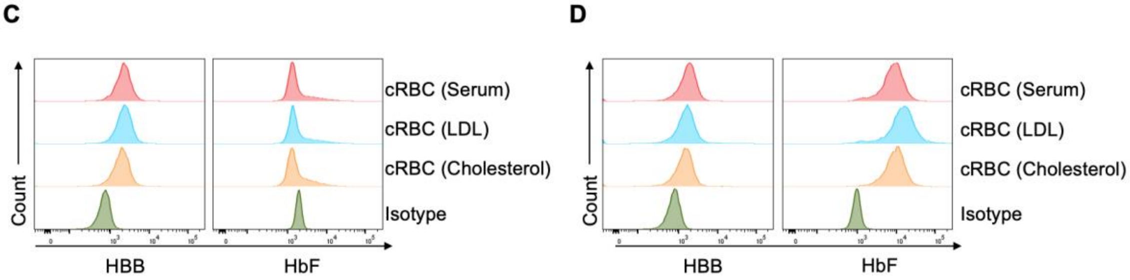

Figure S11. Comparations of cultured reticulocytes and native RBCs. A) Representative bright field, Giemsa, and scanning electron microscopy (SEM) images of native RBCs and differentiated PBMC-derived erythroblasts after 8 days of LDL-supplemented terminal maturation. Scale bar in bright field and Giemsa, 20 μm; scale bar in SEM, 2 μm. B) Red blood cell indices and enzyme activities in serum-cultured RBCs (serum cRBCs), LDL-cultured RBCs (LDL cRBCs), cholesterol-cultured RBCs (cholesterol cRBCs) and native RBCs. MCV, mean corpuscular volume; MCH, mean corpuscular hemoglobin; MCHC, mean corpuscular hemoglobin concentration; PK, pyruvate kinase; ATP, adenosine triphosphate. C-D) Flow cytometric analysis

of adult hemoglobin β (HBB) and fetal hemoglobin (HbF) in cRBCs cultured from PBMC-derived C) or CBMC-derived D) erythroblasts in the optimized culture system supplemented with serum, LDL, or cholesterol.

Supplemental Table 1. Proteins identified in selected fractions by LC-MS/MS analysis (Fraction 9)

Protein FDR Confidence: Combined	Accession	Exp. q-value: Combined	Sum PEP Score	Coverage [%]	# Peptides	# PSMs	# Unique Peptides	# Protein Groups	# AAs	MW [kDa]	calc. pI	Score Sequest HT: Sequest HT	# Peptides (by Search Engine): Sequest HT
High	P04114	0	1164.817	51	197	452	197	1	4563	515.3	7.05	1299.75	197
High	P01024	0	1096.032	76	128	735	128	1	1663	187	6.4	2295.08	128
High	P01023	0	864.677	65	76	595	68	1	1474	163.2	6.46	2048.26	76
High	P0C0L5	0	813.144	53	78	392	4	1	1744	192.6	7.27	1397.94	78
High	P0C0L4	0	778.908	52	76	388	2	1	1744	192.7	7.08	1375.74	76
High	P08603	0	403.047	48	53	194	50	1	1231	139	6.61	580.11	53
High	P01031	0	393.235	42	65	168	65	1	1676	188.2	6.52	480.39	65
High	P02768	0	384.659	79	49	220	49	1	609	69.3	6.28	723.04	49
High	P02751	0	368.75	31	51	122	51	1	2386	262.5	5.71	394.52	51
High	P02647	0	346.845	82	41	251	41	1	267	30.8	5.76	728.53	41
High	P69905	0	50.467	56	6	20	6	1	142	15.2	8.68	63.73	6
High	P19823	0	297.813	40	35	157	35	1	946	106.4	6.86	524.39	35
High	P0DOX5	0	289.743	51	19	213	10	1	449	49.3	8.72	742.78	19
High	P68871	0	88.644	90	13	31	13	1	147	16	7.28	96.35	13
High	P19827	0	220.307	37	27	141	27	1	911	101.3	6.79	407.57	27
High	P01009	0	211.071	64	28	122	28	1	418	46.7	5.59	363.96	28
High	P01008	0	177.908	48	26	80	26	1	464	52.6	6.71	249.98	26
High	P10909	0	65.15	23	11	31	11	1	449	52.5	6.27	82.92	11
High	P01860	0	170.041	53	15	135	6	1	377	41.3	7.9	426.98	15
High	P00450	0	168.069	35	28	54	28	1	1065	122.1	5.72	156.11	28
High	P0DOX2	0	157.725	47	17	107	9	1	455	48.9	6.81	378.94	17
High	P01861	0	154.039	59	14	74	7	1	327	35.9	7.36	258.35	14
High	P01859	0	151.355	53	13	124	6	1	326	35.9	7.59	350.81	13
High	P01834	0	147.117	82	8	224	2	1	107	11.8	6.52	894.97	8
High	P05155	0	171.311	30	19	109	19	1	500	55.1	6.55	332.57	19
High	P0DOX7	0	144.344	55	9	200	2	1	214	23.4	7.17	776.77	9
High	P10643	0	117.954	33	23	42	23	1	843	93.5	6.48	117.75	23
High	P00734	0	113.058	27	14	40	14	1	622	70	5.9	126.86	14
High	P01871	0	103.536	42	15	74	1	1	453	49.4	6.77	221.48	15
High	P20742	0	100.481	13	16	77	8	1	1482	163.8	6.38	216.25	16
High	P00736	0	94.947	32	15	31	14	1	705	80.1	6.21	87.56	15
High	P0DOX6	0	92.191	28	14	71	1	1	576	63.4	7.87	201.39	14
High	P03952	0	61.222	35	17	30	17	1	638	71.3	8.22	60.82	17
High	P04003	0	88.054	37	19	34	19	1	597	67	7.3	84.43	19
High	P27169	0	85.939	54	13	42	13	1	355	39.7	5.22	105.78	13
High	P04220	0	84.104	39	12	51	1	1	391	43	5.24	159.03	12
High	P02649	0	81.45	55	16	31	16	1	317	36.1	5.73	89.31	16
High	P06727	0	71.491	47	17	27	17	1	396	45.4	5.38	63.34	17
High	P02766	0	71.306	54	8	20	8	1	147	15.9	5.76	72.53	8
High	P01042	0	70.91	23	13	27	13	1	644	71.9	6.81	72.19	13
High	P02760	0	19.603	28	7	14	7	1	352	39	6.25	31.84	7
High	P04264	0	64.885	23	14	25	12	1	644	66	8.12	72.84	14
High	P22792	0	63.136	28	12	30	12	1	545	60.5	5.99	86.6	12
High	P08697	0	61.591	29	10	16	10	1	491	54.5	6.29	49.39	10
High	P04196	0	61.299	28	12	26	12	1	525	59.5	7.5	65.25	12
High	P01876	0	224.749	58	18	245	11	1	353	37.6	6.51	876.18	18
High	P0DOX3	0	61.187	39	13	24	13	1	512	56.2	8.02	51.88	13
High	P09871	0	59.258	20	11	20	11	1	688	76.6	4.96	60.02	11
High	P02652	0	57.98	69	9	17	9	1	100	11.2	6.62	57.8	9
High	P0DOY2	0	57.444	79	6	56	2	1	106	11.3	7.24	185.58	6
High	P0DOX8	0	55.453	39	6	50	3	1	216	22.8	6.76	182.02	6
High	P15169	0	54.864	31	9	18	9	1	458	52.3	7.34	58.55	9
High	P04004	0	54.147	27	11	31	11	1	478	54.3	5.8	90.17	11
High	O75882	0	53.224	10	12	24	12	1	1429	158.4	7.31	62.46	12
High	P35527	0	53.181	25	9	12	9	1	623	62	5.24	43.64	9
High	Q14624	0	50.478	14	10	22	10	1	930	103.3	6.98	61.29	10
High	P00739	0	144.848	45	19	202	4	1	348	39	7.09	552.83	19
High	O14791	0	48.63	23	7	16	7	1	398	43.9	5.81	43.89	7
High	P02787	0	45.215	17	11	19	11	1	698	77	7.12	29.19	11
High	P13671	0	44.909	16	9	9	9	1	934	104.7	6.76	28.82	9
High	P06396	0	42.559	16	8	12	8	1	782	85.6	6.28	31.57	8
High	P07225	0	40.925	16	9	17	9	1	676	75.1	5.67	43.97	9
High	Q06033	0	38.496	13	7	15	7	1	890	99.8	5.74	37.97	7
High	P05546	0	35.974	24	12	22	12	1	499	57	6.9	48.04	12
High	P35858	0	34.368	22	10	13	10	1	605	66	6.79	35.62	10
High	P05156	0	33.845	17	7	11	7	1	583	65.7	7.5	22.58	7
High	P35908	0	32.636	19	8	12	6	1	639	65.4	8	29.84	8
High	P13645	0	31.885	22	9	11	9	1	584	58.8	5.21	20.65	9

High	P02743	0	31.725	27	7	15	7	1	223	25.4	6.54	41.66	7
High	P05090	0	30.755	38	9	16	9	1	189	21.3	5.15	35.06	9
High	Q03591	0	30.349	24	7	14	3	1	330	37.6	7.39	31.61	7
High	P06276	0	29.683	12	5	9	5	1	602	68.4	7.42	20.49	5
High	A0M8Q6	0	28.283	42	3	45	1	1	106	11.2	8.29	154.43	3
High	P02747	0	23.966	25	5	10	5	1	245	25.8	8.41	25.44	5
High	Q96PD5	0	21.847	10	4	8	4	1	576	62.2	7.55	23.16	4
High	P60709	0	20.202	19	6	9	6	1	375	41.7	5.48	20.74	6
High	P00738	0	317.739	70	32	301	17	1	406	45.2	6.58	894.69	32
High	P02746	0	18.997	11	2	5	2	1	253	26.7	8.63	15.99	2
High	O95445	0	17.495	44	6	13	6	1	188	21.2	6.01	24.41	6
High	A0A075B6R2	0	17.041	38	3	14	2	1	117	12.8	9.6	36.14	3
High	O43866	0	17.032	24	6	7	6	1	347	38.1	5.47	14.03	6
High	P01780	0	16.796	32	4	18	2	1	117	12.9	6.57	42.45	4
High	P06312	0	16.575	30	3	9	3	1	121	13.4	5.25	22.86	3
High	P01782	0	16.546	35	3	9	2	1	118	12.9	7.08	22.74	3
High	P55058	0	16.476	12	4	4	4	1	493	54.7	7.01	9.59	4
High	P01019	0	16.417	11	4	6	4	1	485	53.1	6.32	15.36	4
High	P29622	0	15.86	18	6	7	6	1	427	48.5	7.75	15.98	6
High	P01597	0	15.323	29	2	7	1	1	117	12.7	8.66	21	2
High	O75636	0	15.227	14	4	7	4	1	299	32.9	6.67	17.18	4
High	Q9NZP8	0	14.955	10	4	7	3	1	487	53.5	7.2	9.35	4
High	P02656	0	14.449	34	2	3	2	1	99	10.8	5.41	9.42	2
High	A0A075B6S5	0	13.988	54	3	5	3	1	117	12.7	8.29	11.79	3
High	P01824	0	13.927	20	2	16	1	1	125	13.9	9.26	40.03	2
High	P01619	0	13.798	28	3	12	3	1	116	12.5	4.96	38.95	3
High	Q08380	0	13.246	7	3	5	3	1	585	65.3	5.27	11.57	3
High	P01593	0	13.187	14	1	4	1	1	117	12.8	4.78	13.65	1
High	A0A0C4DH72	0	12.62	30	2	7	1	1	117	12.7	8.29	19.9	2
High	A0A0B4J1X5	0	12.285	25	3	13	1	1	117	12.8	8.66	31.98	3
High	P22891	0	12.145	9	5	7	5	1	400	44.7	5.97	7.83	5
High	A0A075B6K4	0	11.268	28	3	4	3	1	115	12.4	4.83	12.29	3
High	P19652	0	11.193	9	2	7	2	1	201	23.6	5.11	16.86	2
High	A0A0A0MRZ8	0	10.958	26	2	3	2	1	115	12.6	5.29	9.97	2
High	P36980	0	10.79	17	3	3	1	1	270	30.6	6.38	7.33	3
High	P00747	0	10.57	4	4	6	4	1	810	90.5	7.24	11.6	4
High	A0A0C4DH38	0	10.496	24	2	7	1	1	117	12.7	8.27	17.67	2
High	P80108	0	10.218	5	3	3	3	1	840	92.3	6.37	5.83	3
High	P0DP03	0	10.174	25	3	7	2	1	117	12.9	8.92	16.6	3
High	P01011	0	9.878	9	3	3	3	1	423	47.6	5.52	7.47	3
High	P01591	0	9.822	22	3	3	3	1	159	18.1	5.24	8.37	3
High	A0A075B6S2	0	9.31	26	3	3	1	1	120	13.1	7.12	7.71	3
High	P0DP01	0	9.031	22	2	5	1	1	117	13	9.17	10.32	2
High	P80748	0	8.856	39	2	6	2	1	117	12.4	5.29	19.44	2
High	P01743	0	8.744	22	2	5	1	1	117	12.9	8.92	10.45	2
High	P02745	0	8.682	13	2	3	2	1	245	26	9.11	11.15	2
High	P43652	0	8.56	5	3	4	3	1	599	69	5.9	4.88	3
High	Q15848	0	8.261	12	2	2	2	1	244	26.4	5.74	4.92	2
High	Q6EMK4	0	8.25	6	3	4	3	1	673	71.7	7.39	6.69	3
High	P35542	0	8.016	33	3	4	3	1	130	14.7	9.07	6.44	3
High	Q96KN2	0	8.004	9	3	3	3	1	507	56.7	5.3	4.24	3
High	Q86YZ3	0	7.752	2	1	1	1	1	2850	282.2	10.04	4.71	1
High	P18428	0	7.296	8	2	2	2	1	481	53.4	6.7	2.56	2
High	P01602	0	7.121	25	2	3	1	1	117	12.8	8.28	7.68	2
High	P23142	0	7.088	4	3	5	3	1	703	77.2	5.22	9.16	3
High	P02749	0	7.055	13	3	3	3	1	345	38.3	7.97	6.08	3
High	P02765	0	6.79	5	2	2	2	1	367	39.3	5.72	4.91	2
High	A0A087WW87	0	6.761	17	2	2	1	1	121	13.3	4.61	5.87	2
High	A0A075B6S6	0	6.668	33	2	2	1	1	120	13.2	7.99	3.51	2
High	P15814	0	6.503	14	2	3	2	1	213	22.9	10.07	8.28	2
High	P00748	0	6.391	3	1	1	1	1	615	67.7	7.74	3.71	1
High	P09172	0	6.285	3	1	1	1	1	617	69	6.42	3.94	1
High	A0A0J9YXX1	0	5.861	17	2	4	1	1	117	12.8	8.28	8.15	2
High	A0A0C4DH31	0	5.78	22	2	2	1	1	117	12.8	8.84	5.65	2
High	A0A0C4DH68	0	5.772	17	2	3	1	1	120	13.1	8.53	6.77	2
High	P22352	0	5.73	10	2	2	2	1	226	25.5	8.13	3.38	2
High	P02655	0	5.643	33	2	4	2	1	101	11.3	4.72	6.05	2
High	P23083	0	5.475	11	2	2	1	1	117	13.1	9.13	3.77	2
High	P01718	0	5.376	33	3	3	3	1	113	12.2	5.01	4.03	3
High	Q04756	0	5.306	3	1	2	1	1	655	70.6	7.24	5.82	1
High	Q9NQ79	0	5.192	3	1	1	1	1	661	71.4	5.12	3.52	1
High	P07358	0	4.967	4	1	1	1	1	591	67	8.13	3.07	1
High	A0A0A0MS15	0	4.753	18	2	4	2	1	119	13	8.62	6.57	2
High	Q92954	0	4.736	2	2	2	2	1	1404	151	9.5	2.37	2
High	A0A0B4J1V2	0	4.68	18	2	2	2	1	119	13.2	8.29	1.98	2
High	A0A0B4J1Y9	0	4.668	9	1	9	1	1	119	13.2	7.85	8.94	1
High	O00391	0	4.487	3	1	1	1	1	747	82.5	8.92	3.62	1
High	P01700	0	4.355	11	1	4	1	1	117	12.3	5.91	11.27	1
High	Q6UXB8	0	4.335	7	2	3	2	1	463	49.4	5.39	4.57	2
High	A0A0B4J1V0	0	4.172	11	1	2	1	1	119	12.9	8.62	5.32	1

High	P02790	0	4.167	7	2	2	2	1	462	51.6	7.02	2.2	2
High	Q14520	0	4.039	4	2	2	2	1	560	62.6	6.54	0	2
High	P01701	0	3.989	14	2	2	2	1	117	12.2	7.03	2.2	2
High	A0A0C4DH34	0	3.757	14	1	2	1	1	117	13.1	9.29	3.84	1
High	P33151	0	3.458	1	1	1	1	1	784	87.5	5.43	2.73	1
High	P02748	0	3.44	2	1	2	1	1	559	63.1	5.59	3.71	1
High	P55056	0	3.365	9	1	1	1	1	127	14.5	8.92	1.99	1
High	P49747	0	3.07	5	2	2	2	1	757	82.8	4.6	0	2
High	Q9Y5Y7	0	2.977	3	1	1	1	1	322	35.2	8.28	1.91	1
High	P22105	0	2.961	0	1	1	1	1	4242	457.9	5.17	2.22	1
High	P13473	0	2.774	2	1	1	1	1	410	44.9	5.63	2.05	1
High	A0A087WSY6	0	2.754	8	1	2	1	1	115	12.5	5.19	4.02	1
High	A0A087WSX0	0	2.726	13	1	1	1	1	123	13.2	7.2	2.34	1
High	P51884	0	2.632	2	1	1	1	1	338	38.4	6.61	2.38	1
High	P05160	0	2.592	2	1	1	1	1	661	75.5	6.39	2	1
High	A0A0C4DH55	0	2.509	8	1	2	1	1	119	13.1	5.94	5.31	1
High	Q9UHG3	0	2.507	7	2	2	2	1	505	56.6	6.18	0	2
High	Q9UK55	0	2.472	5	1	1	1	1	444	50.7	8.27	2.63	1
High	P00751	0	2.46	1	1	1	1	1	764	85.5	7.06	2.12	1
High	P02654	0	2.28	11	1	1	1	1	83	9.3	8.47	1.73	1
High	P20851	0	2.222	4	1	1	1	1	252	28.3	5.14	1.93	1
High	P01714	0	2.161	8	1	1	1	1	112	12	4.96	2.37	1
High	P12259	0	2.096	1	1	1	1	1	2224	251.5	6.05	1.82	1
High	A0A075B6I9	0	2.052	8	1	1	1	1	117	12.5	7.2	2.19	1
High	A0A075B6Q5	0	2.033	9	1	1	1	1	118	12.9	7.85	2.54	1
High	Q9HDC9	0.008	1.713	2	1	1	1	1	416	46.5	6.16	0	1
Medium	P04070	0.016	1.584	4	1	1	1	1	461	52	6.28	0	1
Medium	P04217	0.015	1.375	1	1	1	1	1	495	54.2	5.86	1.73	1
Medium	B2RTY4	0.026	1.132	1	1	1	1	1	2548	292.5	8.88	0	1
Medium	Q6IB77	0.038	1.06	13	1	1	1	1	296	33.9	8.16	0	1
Medium	A0A0B4J1U7	0.037	1.041	6	1	5	1	1	121	13.5	9.2	7.61	1
Medium	P08519	0.037	1.025	8	1	1	1	1	4548	501	5.88	0	1
Medium	Q13554	0.037	1.019	5	1	1	1	1	666	72.6	7.27	0	1
Low	P22455	0.067	0.919	1	1	1	1	1	802	87.9	6.81	2.02	1
Low	Q9Y2F5	0.07	0.895	0	1	1	1	1	2266	247.7	5.48	1.69	1
Low	P11168	0.076	0.886	2	1	1	1	1	524	57.5	7.97	1.84	1
Low	Q15582	0.076	0.878	2	1	1	1	1	683	74.6	7.71	1.66	1
Low	Q5SYB0	0.079	0.854	0	1	1	1	1	1578	173.3	5.25	0	1

Supplemental Table 1. Proteins identified in selected fractions by LC-MS/MS analysis (Fraction 12)

Protein FDR Confidence: Combined	Accession	Exp. q-value: Combined	Sum PEP Score	Coverage [%]	# Peptides	# PSMs	# Unique Peptides	# Protein Groups	# AAs	MW [kDa]	calc. pI	Sequest HT: Sequest HT	Search Engine): Sequest HT
High	P01024	0	1071.924	77	129	954	129	1	1663	187	6.4	3044.15	129
High	P0DOX5	0	364.88	53	26	397	13	1	449	49.3	8.72	1447.13	26
High	P01834	0	146.354	86	10	288	3	1	107	11.8	6.52	1285.09	10
High	P0DOX7	0	140.724	57	11	260	3	1	214	23.4	7.17	1149.17	11
High	P0C0L5	0	558.937	49	72	238	3	1	1744	192.6	7.27	835.04	72
High	P0C0L4	0	556.672	49	73	238	4	1	1744	192.7	7.08	826.61	73
High	P02768	0	391.748	77	50	219	50	1	609	69.3	6.28	770.24	50
High	P01859	0	217.816	70	22	214	11	1	326	35.9	7.59	687.36	22
High	P01860	0	174.321	57	20	194	7	1	377	41.3	7.9	616.23	20
High	P00450	0	473.773	51	44	170	44	1	1065	122.1	5.72	649	44
High	P00738	0	203.336	63	26	169	13	1	406	45.2	6.58	497.71	26
High	P01023	0	303.165	46	50	144	50	1	1474	163.2	6.46	429.85	50
High	P02647	0	238.195	81	35	133	35	1	267	30.8	5.76	423.35	35
High	P01861	0	191.587	61	16	129	8	1	327	35.9	7.36	457.26	16
High	P01876	0	154.801	55	16	128	10	1	353	37.6	6.51	470.29	16
High	P00739	0	80.292	41	17	101	4	1	348	39	7.09	257.34	17
High	P19823	0	221.139	37	31	98	31	1	946	106.4	6.86	345.04	31
High	P05155	0	118.206	30	17	88	17	1	500	55.1	6.55	262.73	17
High	P19827	0	134.293	31	22	87	22	1	911	101.3	6.79	260.04	22
High	P00747	0	164.848	43	32	76	32	1	810	90.5	7.24	230.8	32
High	P0DOY2	0	76.52	86	9	72	4	1	106	11.3	7.24	242.78	9
High	P01031	0	169.081	32	43	70	43	1	1676	188.2	6.52	198.21	43
High	P0DOX2	0	104.921	45	15	70	8	1	455	48.9	6.81	225.74	15
High	P08603	0	153.403	35	32	66	30	1	1231	139	6.61	181.77	32
High	P06727	0	151.249	67	27	65	27	1	396	45.4	5.38	205.23	27
High	P01009	0	132.593	56	21	65	21	1	418	46.7	5.59	211.66	21
High	Q14624	0	127.092	31	24	60	24	1	930	103.3	6.98	181.7	24
High	P0DOX8	0	64.165	46	9	57	4	1	216	22.8	6.76	204.62	9
High	P02790	0	94.737	48	17	56	17	1	462	51.6	7.02	155.8	17
High	P05546	0	87.669	42	18	53	18	1	499	57	6.9	156.66	18
High	A0M8Q6	0	33.919	46	4	53	1	1	106	11.2	8.29	186.38	4
High	P04114	0	126.195	13	46	52	46	1	4563	515.3	7.05	93.13	46
High	P01619	0	37.154	47	5	52	2	1	116	12.5	4.96	165.81	5
High	P00751	0	112.854	27	23	50	23	1	764	85.5	7.06	141.55	23
High	P02787	0	129.453	50	28	48	28	1	698	77	7.12	124.5	28
High	P01042	0	98.352	29	18	45	18	1	644	71.9	6.81	133.85	18
High	A0A0C4DH25	0	19.467	28	4	44	1	1	116	12.5	4.59	137.26	4
High	P13671	0	128.366	31	24	43	24	1	934	104.7	6.76	135.29	24
High	P01008	0	92.3	40	19	43	19	1	464	52.6	6.71	99.89	19
High	P04196	0	79.65	30	14	42	14	1	525	59.5	7.5	126.29	14
High	P07358	0	85.526	31	18	38	18	1	591	67	8.13	113.26	18
High	P35858	0	84.126	33	16	37	16	1	605	66	6.79	115.27	16
High	P01011	0	76.339	37	18	37	18	1	423	47.6	5.52	107.25	18
High	P06396	0	113.646	41	21	36	21	1	782	85.6	6.28	116.12	21
High	P00734	0	102.797	33	16	36	16	1	622	70	5.9	120.72	16
High	P02749	0	74.247	48	13	35	13	1	345	38.3	7.97	110.71	13
High	P10643	0	80.645	30	22	34	22	1	843	93.5	6.48	91.46	22
High	P08697	0	92.682	34	14	32	14	1	491	54.5	6.29	102.15	14
High	P10909	0	61.685	29	14	30	14	1	449	52.5	6.27	80.77	14
High	P01871	0	45.898	37	12	29	2	1	453	49.4	6.77	74.01	12
High	P0DOX6	0	39.985	23	11	29	1	1	576	63.4	7.87	69.36	11
High	P04217	0	59.171	36	12	28	12	1	495	54.2	5.86	86.13	12
High	P02748	0	56.327	29	13	27	13	1	559	63.1	5.59	73.13	13
High	P51884	0	51.293	36	11	24	11	1	338	38.4	6.61	65.25	11
High	P04004	0	40.064	19	9	24	9	1	478	54.3	5.8	67.32	9
High	Q96PD5	0	50.849	22	8	21	8	1	576	62.2	7.55	66.74	8
High	P27169	0	47.192	36	9	21	9	1	355	39.7	5.22	58.95	9
High	P29622	0	39.735	34	12	21	12	1	427	48.5	7.75	44.46	12
High	P01780	0	20.573	40	5	21	2	1	117	12.9	6.57	47.02	5
High	P07357	0	52.811	24	11	20	11	1	584	65.1	6.47	60.05	11
High	P43652	0	48.602	22	12	20	12	1	599	69	5.9	51.26	12
High	Q06033	0	43.105	13	10	20	10	1	890	99.8	5.74	47.76	10
High	P05156	0	50.622	21	11	19	11	1	583	65.7	7.5	48.25	11
High	P00748	0	57.685	20	9	17	9	1	615	67.7	7.74	59.58	9
High	P02649	0	56.685	42	11	17	11	1	317	36.1	5.73	47.65	11
High	P02766	0	47.655	69	7	17	7	1	147	15.9	5.76	57.94	7
High	P02652	0	47.575	69	9	17	9	1	100	11.2	6.62	59.27	9
High	P06681	0	23.742	13	9	16	9	1	752	83.2	7.42	31.51	9
High	P80108	0	44.593	20	11	15	11	1	840	92.3	6.37	51.27	11
High	P02743	0	23.999	23	6	15	6	1	223	25.4	6.54	37.07	6
High	A0A0B4J1X5	0	15.452	32	4	15	1	1	117	12.8	8.66	34.28	4
High	A0A0C4DH38	0	11.279	30	3	15	2	1	117	12.7	8.27	27.55	3
High	P07360	0	49.275	65	9	14	9	1	202	22.3	8.31	44.19	9
High	P02751	0	45.822	7	10	14	10	1	2386	262.5	5.71	37.64	10
High	P06312	0	17.797	36	4	14	4	1	121	13.4	5.25	37.75	4
High	P06331	0	17.223	37	3	14	2	1	123	13.8	9.33	43.05	3
High	P68871	0	32.469	69	8	13	8	1	147	16	7.28	38.66	8
High	P02760	0	18.279	20	7	13	7	1	352	39	6.25	29.09	7

High	P04264	0	30.958	16	9	13	9	1	644	66	8.12	33.05	9
High	P07225	0	27	13	8	12	8	1	676	75.1	5.67	27.43	8
High	A0A075B6S5	0	16.616	29	2	12	2	1	117	12.7	8.29	42.05	2
High	P02765	0	28.864	27	6	11	6	1	367	39.3	5.72	30.45	6
High	P01782	0	13.969	26	3	11	1	1	118	12.9	7.08	25.93	3
High	P03952	0	23.348	15	8	10	8	1	638	71.3	8.22	15.05	8
High	P05160	0	24.043	15	7	10	7	1	661	75.5	6.39	20.38	7
High	P01597	0	19.858	29	2	10	1	1	117	12.7	8.66	32.83	2
High	A0A0C4DH72	0	16.49	40	3	10	1	1	117	12.7	8.29	29.98	3
High	A0A075B6R2	0	15.102	38	3	10	2	1	117	12.8	9.6	33.32	3
High	P13645	0	28.928	13	6	9	5	1	584	58.8	5.21	23.32	6
High	Q03591	0	24.444	25	7	9	5	1	330	37.6	7.39	27.98	7
High	P01624	0	18.803	26	2	9	1	1	115	12.5	5.19	32.8	2
High	A0A0C4DH73	0	13.876	29	2	9	1	1	117	12.6	8.31	28.95	2
High	P69905	0	26.451	35	4	8	4	1	142	15.2	8.68	25.32	4
High	P60709	0	16.81	15	4	8	4	1	375	41.7	5.48	18.82	4
High	P04433	0	15.011	52	3	8	3	1	115	12.6	4.96	25.69	3
High	P01764	0	12.706	22	3	8	2	1	117	12.6	8.28	23.16	3
High	P01766	0	10.377	19	2	8	1	1	116	12.5	7.08	19.26	2
High	A0A0B4J1Y9	0	6.496	18	2	8	2	1	119	13.2	7.85	13.88	2
High	P06310	0	19.128	42	4	7	3	1	120	13.2	8.51	21.33	4
High	6	0	18.916	26	2	7	1	1	115	12.5	5.19	27.35	2
High	P35527	0	17.554	18	6	7	5	1	623	62	5.24	16.34	6
High	P02774	0	16.001	15	6	7	6	1	474	52.9	5.54	20.57	6
High	P09871	0	14.985	9	5	7	5	1	688	76.6	4.96	19.63	5
High	A0A075B6S2	0	12.742	26	3	7	1	1	120	13.1	7.12	17.18	3
High	7	0	12.338	17	2	7	1	1	121	13.3	4.61	22.22	2
High	P01742	0	10.029	28	3	7	2	1	117	12.7	9.17	19.48	3
High	A0A0C4DH34	0	6.239	21	2	7	2	1	117	13.1	9.29	10.29	2
High	P04278	0	17.934	24	5	6	5	1	402	43.8	6.71	16.77	5
High	P01602	0	17.441	25	2	6	1	1	117	12.8	8.28	20.25	2
High	P01599	0	14.931	24	3	6	2	1	117	12.8	8.68	17.51	3
High	Q16610	0	13.767	13	6	6	6	1	540	60.6	6.71	12.46	6
High	P23083	0	12.842	22	3	6	2	1	117	13.1	9.13	15.52	3
High	A0A0A0MS15	0	8.977	28	3	6	3	1	119	13	8.62	15.02	3
High	P80748	0	7.544	14	1	6	1	1	117	12.4	5.29	22.43	1
High	A0A0C4DH29	0	5.713	22	2	6	1	1	117	13	9.55	15.64	2
High	P01743	0	5.627	22	2	6	1	1	117	12.9	8.92	16.36	2
High	P01019	0	17.695	10	3	5	3	1	485	53.1	6.32	17.12	3
High	O00391	0	15.807	10	5	5	5	1	747	82.5	8.92	12.91	5
High	A0A0B4J1V2	0	14.621	41	3	5	3	1	119	13.2	8.29	11.74	3
High	Q9NZP8	0	14.04	13	5	5	4	1	487	53.5	7.2	11.8	5
High	O14791	0	13.815	15	4	5	4	1	398	43.9	5.81	9.91	4
High	P22792	0	13.011	9	3	5	3	1	545	60.5	5.99	15.87	3
High	A0A075B6K4	0	12.282	28	3	5	2	1	115	12.4	4.83	15.31	3
High	O75882	0	12.047	3	3	5	3	1	1429	158.4	7.31	13.55	3
High	O95445	0	4.408	14	3	5	3	1	188	21.2	6.01	9.88	3
High	P19652	0	15.153	18	3	4	2	1	201	23.6	5.11	12.99	3
High	Q96KN2	0	12.618	11	4	4	4	1	507	56.7	5.3	11.61	4
High	P04180	0	10.017	11	3	4	3	1	440	49.5	6.11	9.75	3
High	P01700	0	8.963	25	2	4	2	1	117	12.3	5.91	10.35	2
High	P0DOX3	0	8.896	6	3	4	3	1	512	56.2	8.02	9.25	3
High	A0A0J9YXX1	0	6.689	17	2	4	1	1	117	12.8	8.28	11.34	2
High	P00736	0	12.608	6	3	3	2	1	705	80.1	6.21	9.35	3
High	P01593	0	10.131	34	2	3	2	1	117	12.8	4.78	10.58	2
High	P01717	0	7.122	19	2	3	1	1	112	12	4.5	7.34	2
High	P04070	0	6.785	12	3	3	3	1	461	52	6.28	4.69	3
High	P00740	0	6.628	4	2	3	2	1	461	51.7	5.47	5.57	2
High	P04003	0	6.234	4	2	3	2	1	597	67	7.3	9.33	2
High	P01701	0	6.199	14	2	3	2	1	117	12.2	7.03	6.47	2
High	A0A0C4DH31	0	6.016	22	2	3	1	1	117	12.8	8.84	10.3	2
High	A0A0A0MT36	0	5.356	18	2	3	2	1	114	12.3	7.28	7.38	2
High	P05090	0	4.711	14	3	3	3	1	189	21.3	5.15	7.38	3
High	P15169	0	4.096	5	2	3	2	1	458	52.3	7.34	7	2
High	P01714	0	4.052	17	2	3	2	1	112	12	4.96	6.76	2
High	A0A075B6I0	0	3.397	13	2	3	2	1	122	12.8	4.55	6.8	2
High	A0A0B4J1V0	0	11.221	13	2	2	2	1	119	12.9	8.62	6.53	2
High	P08519	0	7.815	0	1	2	1	1	4548	501	5.88	6.31	1
High	P02656	0	6.833	27	2	2	2	1	99	10.8	5.41	4.92	2
High	P00742	0	6.709	4	1	2	1	1	488	54.7	5.94	5.51	1
High	P02741	0	4.519	9	2	2	2	1	224	25	5.63	4.49	2
High	A0A0C4DH68	0	4.441	17	2	2	1	1	120	13.1	8.53	4.28	2
High	P05452	0	4.31	6	1	2	1	1	202	22.5	5.67	5.69	1
High	P43251	0	3.623	5	2	2	2	1	543	61.1	6.25	4.37	2
High	P01706	0	3.078	13	2	2	1	1	119	12.6	7.24	4.89	2
High	P02763	0	2.985	12	2	2	1	1	201	23.5	5.02	5.07	2
High	P02747	0	2.814	4	1	2	1	1	245	25.8	8.41	4.56	1
High	A0A075B6I9	0.004	2.182	8	1	2	1	1	117	12.5	7.2	4.63	1
High	P14151	0.004	2.133	2	1	2	1	1	372	42.2	6.6	3.74	1
High	Q9ERE9	0.008	1.774	2	1	2	1	1	830	89.8	4.42	5.09	1

Medium	Q6P5R6	0.023	1.282	6	1	2	1	1	122	14.6	9.38	1.65	1
Medium	Q77PU6	0.045	0.991	2	1	2	1	1	1078	121.4	5.08	0	1
High	P01601	0	5.666	14	1	1	1	1	117	12.7	7.74	3.39	1
High	Q12805	0	4.075	5	1	1	1	1	493	54.6	5.07	3.36	1
High	Q9UK55	0	3.573	4	1	1	1	1	444	50.7	8.27	2.69	1
High	P01591	0	3.474	8	1	1	1	1	159	18.1	5.24	2.26	1
High	Q15582	0	3.429	2	1	1	1	1	683	74.6	7.71	2.37	1
High	Q86YZ3	0	3.394	2	1	1	1	1	2850	282.2	10.04	2.83	1
High	P18428	0	3.208	4	1	1	1	1	481	53.4	6.7	2.68	1
High	A0A0B4J1U3	0	3.158	14	1	1	1	1	117	12.5	4.78	3.02	1
High	P02745	0	2.883	9	1	1	1	1	245	26	9.11	3.46	1
High	P06276	0.004	2.795	3	1	1	1	1	602	68.4	7.42	2.79	1
High	Q04756	0.004	2.753	2	1	1	1	1	655	70.6	7.24	2.07	1
High	P05543	0.004	2.709	7	1	1	1	1	415	46.3	6.3	2.17	1
High	P33151	0.004	2.637	1	1	1	1	1	784	87.5	5.43	2.76	1
High	A0A075B6J9	0.004	2.313	11	1	1	1	1	118	12.4	4.82	1.62	1
High	A0A075B6Q5	0.004	2.293	9	1	1	1	1	118	12.9	7.85	2.42	1
High	P08185	0.004	1.94	4	1	1	1	1	405	45.1	6.04	2.64	1
High	Q9NQ79	0.008	1.665	3	1	1	1	1	661	71.4	5.12	2.2	1
Medium	P01704	0.02	1.56	13	1	1	1	1	120	12.6	6.49	2.42	1
Medium	A0A075B6I4	0.02	1.512	9	1	1	1	1	117	12.4	8.03	2.56	1
Medium	Q4G0P3	0.023	1.311	0	1	1	1	1	5121	575.5	6.06	1.64	1
Medium	Q6EMK4	0.027	1.181	2	1	1	1	1	673	71.7	7.39	0	1
Medium	P26927	0.046	1.037	1	1	1	1	1	711	80.3	7.68	0	1
Low	Q06730	0.053	0.979	2	1	1	1	1	810	94.3	8.07	2.1	1
Low	Q79670	0.052	0.975	1	1	1	1	1	876	99.2	8.68	0	1
Low	P49908	0.067	0.886	2	1	1	1	1	381	43.2	7.87	0	1

Supplemental Table 1. Proteins identified in selected fractions by LC-MS/MS analysis (Fraction 15)

Protein FDR Confidence: Combined	Accession	Exp. q-value: Combined	Sum PEP Score	Coverage [%]	# Peptides	# PSMs	# Unique Peptides	# Protein Groups	# AAs	MW [kDa]	calc. pI	Score Sequest HT: Sequest HT	# Peptides (by Search Engine): Sequest HT
High	P02768	0	1352.346	92	97	1532	97	1	609	69.3	6.28	6129.4	97
High	P02787	0	932.398	83	76	507	76	1	698	77	7.12	2006.69	76
High	P01024	0	762.667	59	106	351	106	1	1663	187	6.4	1172.54	106
High	P0DOX5	0	300.212	52	23	204	11	1	449	49.3	8.72	815.99	23
High	P01009	0	296.952	66	33	204	33	1	418	46.7	5.59	729.94	33
High	P02790	0	277.847	65	31	175	31	1	462	51.6	7.02	630.06	31
High	P01834	0	136.724	82	9	138	3	1	107	11.8	6.52	591.03	9
High	P00450	0	463.616	49	46	113	46	1	1065	122.1	5.72	474.02	46
High	P0DOX7	0	117.897	55	9	107	3	1	214	23.4	7.17	457.32	9
High	P01859	0	191.122	57	18	99	7	1	326	35.9	7.59	339.51	18
High	P01011	0	167.109	44	24	97	24	1	423	47.6	5.52	314.89	24
High	P01860	0	144.991	56	19	95	7	1	377	41.3	7.9	303.64	19
High	P00751	0	239.258	43	39	84	39	1	764	85.5	7.06	269.96	39
High	P04217	0	174.799	52	20	84	20	1	495	54.2	5.86	308.6	20
High	P00747	0	206.058	53	36	72	36	1	810	90.5	7.24	245.53	36
High	P01861	0	146.188	56	15	72	7	1	327	35.9	7.36	265.6	15
High	P0C0L5	0	246.336	28	39	68	2	1	1744	192.6	7.27	257.28	39
High	P0C0L4	0	226.049	28	39	66	2	1	1744	192.7	7.08	241.44	39
High	P01008	0	134.982	44	24	64	24	1	464	52.6	6.71	198.81	24
High	P02647	0	146.567	67	26	60	26	1	267	30.8	5.76	197.5	26
High	P02749	0	167.576	51	17	54	17	1	345	38.3	7.97	211.73	17
High	P43652	0	134.45	37	24	54	24	1	599	69	5.9	154.5	24
High	P00738	0	116.326	50	23	51	23	1	406	45.2	6.58	146.5	23
High	P06396	0	183.95	50	27	47	27	1	782	85.6	6.28	176.56	27
High	Q14624	0	168.43	38	29	46	29	1	930	103.3	6.98	163.31	29
High	P02774	0	145.351	49	21	43	21	1	474	52.9	5.54	153.9	21
High	P01042	0	130.169	26	20	43	20	1	644	71.9	6.81	150.98	20
High	P02766	0	146.811	69	11	42	11	1	147	15.9	5.76	186.99	11
High	P02765	0	125.969	37	13	42	13	1	367	39.3	5.72	158.92	13
High	P0DOY2	0	79.638	86	8	37	4	1	106	11.3	7.24	136.16	8
High	P04196	0	83.977	35	17	35	17	1	525	59.5	7.5	101.28	17
High	P02748	0	94.66	29	15	33	15	1	559	63.1	5.59	102.49	15
High	P0DOX8	0	66.35	42	7	33	3	1	216	22.8	6.76	124.56	7
High	P01876	0	65.702	48	12	33	7	1	353	37.6	6.51	126.08	12
High	Q96PD5	0	105.967	44	15	27	15	1	576	62.2	7.55	91.78	15
High	P06727	0	76.826	53	19	27	19	1	396	45.4	5.38	81.35	19
High	P19823	0	73.257	22	15	25	15	1	946	106.4	6.86	76.63	15
High	P08697	0	116.011	35	13	24	13	1	491	54.5	6.29	101.86	13
High	P01019	0	87.877	26	11	23	11	1	485	53.1	6.32	85.38	11
High	P02763	0	42.884	31	8	23	4	1	201	23.5	5.02	73.04	8
High	P00748	0	55.554	21	11	18	11	1	615	67.7	7.74	56.72	11
High	P04004	0	48.436	25	11	18	11	1	478	54.3	5.8	50.99	11
High	P51884	0	46.598	30	10	18	10	1	338	38.4	6.61	46.16	10
High	P05546	0	29.14	16	9	18	9	1	499	57	6.9	47.05	9
High	P19827	0	64.52	15	11	16	11	1	911	101.3	6.79	56.62	11
High	P0DOX2	0	38.991	29	10	15	4	1	455	48.9	6.81	50.99	10
High	P35858	0	59.187	26	11	14	11	1	605	66	6.79	47.3	11
High	P00734	0	52.648	24	9	14	9	1	622	70	5.9	49.88	9
High	P05155	0	42.235	18	9	14	9	1	500	55.1	6.55	46.13	9
High	P08185	0	41.021	20	7	14	7	1	405	45.1	6.04	50.19	7
High	P02753	0	32.523	29	6	14	6	1	201	23	6.07	39.07	6
High	P19652	0	34.968	28	7	12	3	1	201	23.6	5.11	30.18	7
High	P01619	0	23.875	47	5	12	5	1	116	12.5	4.96	40.2	5
High	P06681	0	25.752	13	7	11	7	1	752	83.2	7.42	29.1	7
High	P02760	0	16.806	18	6	10	6	1	352	39	6.25	20.06	6
High	P05543	0	27.756	26	7	9	7	1	415	46.3	6.3	20.67	7
High	P29622	0	25.512	23	8	9	8	1	427	48.5	7.75	23.06	8
High	P02750	0	21.982	26	7	9	7	1	347	38.2	6.95	23.34	7
High	P02649	0	30.44	31	8	8	8	1	317	36.1	5.73	19.56	8
High	P07358	0	26.79	20	8	8	8	1	591	67	8.13	24.12	8
High	P05156	0	26.028	10	5	8	5	1	583	65.7	7.5	22.25	5
High	P01031	0	24.865	6	8	8	8	1	1676	188.2	6.52	21.46	8
High	P02652	0	24.119	47	6	8	6	1	100	11.2	6.62	16.75	6
High	P07357	0	26.158	14	6	7	6	1	584	65.1	6.47	21.31	6
High	Q14520	0	13.181	9	4	7	4	1	560	62.6	6.54	14.99	4
High	Q96IY4	0	8.418	11	5	6	5	1	423	48.4	7.71	11.82	5
High	P10909	0	16.248	13	4	5	4	1	449	52.5	6.27	12.27	4
High	P36955	0	14.851	12	5	5	5	1	418	46.3	6.38	13.97	5

High	P27169	0	9.126	9	3	5	3	1	355	39.7	5.22	14.3	3
High	A0A0A0MS15	0	6.751	17	3	5	1	1	119	13	8.62	10.18	3
High	P18428	0	20.304	7	3	4	3	1	481	53.4	6.7	12.69	3
High	P13671	0	19.275	6	3	4	3	1	934	104.7	6.76	14.89	3
High	P01780	0	7.211	23	3	4	2	1	117	12.9	6.57	10.17	3
High	A0A0B4J1V0	0	4.764	15	3	4	1	1	119	12.9	8.62	7.36	3
High	P20742	0	3.71	1	1	4	1	1	1482	163.8	6.38	1.66	1
Medium	A0A0B4J1U7	0.049	0.715	6	1	4	1	1	121	13.5	9.2	6.8	1
High	A0A087WW87	0	16.861	17	1	3	1	1	121	13.3	4.61	10.08	1
High	P25311	0	12.101	14	2	3	2	1	298	34.2	6.05	14.28	2
High	P07360	0	10.545	19	3	3	3	1	202	22.3	8.31	9.08	3
High	P02743	0	10.013	14	3	3	3	1	223	25.4	6.54	8.06	3
High	Q96KN2	0	8.279	9	3	3	3	1	507	56.7	5.3	5.57	3
High	P04264	0	7.535	4	2	3	2	1	644	66	8.12	9.3	2
High	P01700	0	6.6	25	2	3	2	1	117	12.3	5.91	8.99	2
High	P06312	0	5.769	20	2	3	2	1	121	13.4	5.25	8.52	2
Medium	Q8NI35	0.026	0.955	0	1	3	1	1	1801	196.2	4.94	3.7	1
High	P10643	0	13.942	4	2	2	2	1	843	93.5	6.48	7.95	2
High	A0A0A0MRZ8	0	10.859	26	2	2	2	1	115	12.6	5.29	7.65	2
High	P01824	0	9.086	20	2	2	2	1	125	13.9	9.26	6.62	2
High	P0DOX6	0	8.246	4	2	2	2	1	576	63.4	7.87	5.44	2
High	P13645	0	7.9	7	2	2	2	1	584	58.8	5.21	6.22	2
High	A0A0C4DH72	0	7.862	30	2	2	2	1	117	12.7	8.29	5.16	2
High	P04180	0	7.082	6	1	2	1	1	440	49.5	6.11	3.46	1
High	P04278	0	6.474	9	2	2	2	1	402	43.8	6.71	6.42	2
High	A0A0C4DH68	0	5.311	17	2	2	2	1	120	13.1	8.53	4.4	2
High	P01743	0	5.165	22	2	2	2	1	117	12.9	8.92	3.42	2
High	P01714	0	4.618	17	2	2	2	1	112	12	4.96	5.23	2
High	P01764	0	4.608	15	2	2	1	1	117	12.6	8.28	5.82	2
High	Q16610	0	3.15	3	1	2	1	1	540	60.6	6.71	5.93	1
High	A0A0C4DH55	0	2.881	8	1	2	1	1	119	13.1	5.94	5.47	1
High	A0A075B6I9	0.006	1.885	8	1	2	1	1	117	12.5	7.2	4.1	1
Medium	Q14376	0.011	1.562	2	1	2	1	1	348	38.3	6.73	4.04	1
Medium	Q66K66	0.011	1.301	3	1	2	1	1	360	39.4	9.92	3.99	1
Medium	P00742	0.011	1.192	2	1	2	1	1	488	54.7	5.94	4.4	1
High	P80748	0	7.186	14	1	1	1	1	117	12.4	5.29	4.27	1
High	P60709	0	6.14	5	1	1	1	1	375	41.7	5.48	4.57	1
High	P01593	0	5.683	14	1	1	1	1	117	12.8	4.78	4.01	1
High	A0A075B6S5	0	5.242	14	1	1	1	1	117	12.7	8.29	3.39	1
High	Q9BXR6	0	5.053	2	1	1	1	1	569	64.4	7.06	3.15	1
High	P05452	0	4.895	6	1	1	1	1	202	22.5	5.67	3.12	1
High	P01599	0	4.763	13	1	1	1	1	117	12.8	8.68	2.93	1
High	P36980	0	4.621	5	1	1	1	1	270	30.6	6.38	2.88	1
High	P05154	0	4.607	3	1	1	1	1	406	45.6	9.26	3.31	1
High	P00740	0	4.315	4	1	1	1	1	461	51.7	5.47	4.87	1
High	P06310	0	4.264	17	1	1	1	1	120	13.2	8.51	3.37	1
High	P08571	0	3.802	5	1	1	1	1	375	40.1	6.23	2.77	1
High	A0A0A0MT36	0	3.42	11	1	1	1	1	114	12.3	7.28	2.36	1
High	P35527	0	3.106	4	1	1	1	1	623	62	5.24	3.32	1
High	A0A075B6K4	0	3.023	7	1	1	1	1	115	12.4	4.83	2.66	1
High	P22792	0	2.898	4	1	1	1	1	545	60.5	5.99	2.65	1
High	P01701	0	2.632	14	1	1	1	1	117	12.2	7.03	3.33	1
High	A0A0C4DH38	0	2.481	11	1	1	1	1	117	12.7	8.27	2.53	1
High	P05160	0	2.296	1	1	1	1	1	661	75.5	6.39	2.44	1
High	A0A0C4DH31	0	2.293	12	1	1	1	1	117	12.8	8.84	2.74	1
High	Q06033	0	2.256	1	1	1	1	1	890	99.8	5.74	1.75	1
High	P04430	0	2.111	14	1	1	1	1	117	12.6	8.16	2.4	1
Medium	P80108	0.011	1.601	4	1	1	1	1	840	92.3	6.37	2.59	1
Medium	P0CG39	0.011	1.556	2	1	1	1	1	1038	117.3	5.97	2	1
Medium	A0A087WSY6	0.011	1.47	8	1	1	1	1	115	12.5	5.19	2.26	1
Medium	P05090	0.011	1.108	4	1	1	1	1	189	21.3	5.15	2.02	1
Medium	Q8IUQ0	0.026	0.934	4	1	1	1	1	354	40.8	7.2	2.41	1
Medium	P33151	0.026	0.933	1	1	1	1	1	784	87.5	5.43	2.26	1
Medium	P08603	0.05	0.753	1	1	1	1	1	1231	139	6.61	2.92	1
Medium	Q8NFC6	0.049	0.715	0	1	1	1	1	3051	330.3	5.08	0	1
Medium	P11168	0.049	0.708	2	1	1	1	1	524	57.5	7.97	1.88	1
Low	P02008	0.053	0.659	5	1	1	1	1	142	15.6	8.21	1.78	1
Low	Q8IV53	0.052	0.623	2	1	1	1	1	801	87	5.67	0	1

Supplemental Table 1. Proteins identified in selected fractions by LC-MS/MS analysis (Fraction 19)

Protein FDR Confidence: Combined	Accession	Exp. q-value: Combined	Sum PEP Score	Coverage [%]	# Peptides	# PSMs	# Unique Peptides	# Protein Groups	# AAs	MW [kDa]	calc. pI	Score Sequest HT: Sequest HT	# Peptides (by Search Engine): Sequest HT
High	P02768	0	926.714	86	84	1691	84	1	609	69.3	6.28	6280.7	84
High	P02787	0	521.15	71	60	296	60	1	698	77	7.12	1105.72	60
High	P01009	0	297.822	70	35	287	35	1	418	46.7	5.59	1063.42	35
High	P01024	0	258.3	40	54	115	54	1	1663	187	6.4	401.6	54
High	P02790	0	141.28	59	21	90	21	1	462	51.6	7.02	311.9	21
High	P02774	0	171.969	61	27	76	27	1	474	52.9	5.54	293.99	27
High	P02766	0	115.519	69	9	57	9	1	147	15.9	5.76	238.26	9
High	P0DOX5	0	68.984	44	13	46	7	1	449	49.3	8.72	143.98	13
High	P01011	0	83.475	37	16	43	16	1	423	47.6	5.52	148.99	16
High	P01008	0	83.586	42	16	41	16	1	464	52.6	6.71	135.95	16
High	P04217	0	89.965	43	12	34	12	1	495	54.2	5.86	139.17	12
High	P00450	0	92.754	25	19	29	19	1	1065	122.1	5.72	100.32	19
High	P06727	0	66.714	49	18	29	18	1	396	45.4	5.38	88.78	18
High	P02647	0	58.089	60	17	28	17	1	267	30.8	5.76	82.51	17
High	P01834	0	40.491	79	5	28	5	1	107	11.8	6.52	102.14	5
High	P02763	0	34.852	34	9	25	4	1	201	23.5	5.02	83.79	9
High	P43652	0	54.316	23	13	23	13	1	599	69	5.9	62.75	13
High	P01859	0	29.001	38	10	22	4	1	326	35.9	7.59	56.06	10
High	P00751	0	55.159	21	15	20	15	1	764	85.5	7.06	62.95	15
High	P01042	0	46.99	17	9	18	9	1	644	71.9	6.81	62.18	9
High	P01019	0	46.116	24	9	18	9	1	485	53.1	6.32	65.16	9
High	P0C0L5	0	58.28	9	9	15	9	1	1744	192.6	7.27	67.94	9
High	P00747	0	35.657	16	10	15	10	1	810	90.5	7.24	44.38	10
High	P01861	0	27.732	29	7	15	1	1	327	35.9	7.36	53.17	7
High	P02765	0	45.714	25	7	14	7	1	367	39.3	5.72	55.31	7
High	P25311	0	36.11	36	9	14	9	1	298	34.2	6.05	50.33	9
High	Q14624	0	35.833	15	11	13	11	1	930	103.3	6.98	45.23	11
High	P02749	0	31.845	32	9	13	9	1	345	38.3	7.97	38.29	9
High	P19652	0	25.197	31	8	13	3	1	201	23.6	5.11	35.41	8
High	P36955	0	24.17	20	7	10	7	1	418	46.3	6.38	31.21	7
High	P02750	0	18.234	24	6	10	6	1	347	38.2	6.95	27.13	6
High	P04264	0	25.632	15	9	9	9	1	644	66	8.12	27.46	9
High	P08185	0	16.877	13	4	8	4	1	405	45.1	6.04	25.79	4
High	P05546	0	15.709	13	6	8	6	1	499	57	6.9	19.14	6
High	P04196	0	17.55	12	5	7	5	1	525	59.5	7.5	22.14	5
High	P08697	0	32.428	16	4	6	4	1	491	54.5	6.29	25.91	4
High	A0M8Q6	0	10.452	32	2	6	2	1	106	11.2	8.29	20.62	2
High	Q96PD5	0	13.603	9	3	5	3	1	576	62.2	7.55	17.6	3
High	P13645	0	12.418	12	4	5	4	1	584	58.8	5.21	13.15	4
High	P29622	0	10.824	11	4	5	4	1	427	48.5	7.75	12.14	4
High	P02649	0	10.698	14	5	5	5	1	317	36.1	5.73	12.7	5
High	P02748	0	10.451	10	5	5	5	1	559	63.1	5.59	11.78	5
High	P00738	0	6.342	9	4	5	4	1	406	45.2	6.58	7.2	4
High	P02760	0	5.352	13	4	5	4	1	352	39	6.25	12.25	4
High	P06396	0	13.717	7	4	4	4	1	782	85.6	6.28	13.77	4
High	P19827	0	12.283	7	4	4	4	1	911	101.3	6.79	14.54	4
High	P05543	0	10.763	12	3	3	3	1	415	46.3	6.3	8.48	3
High	P04004	0	9.751	5	2	3	2	1	478	54.3	5.8	8.92	2
High	Q14520	0	7.377	6	3	3	3	1	560	62.6	6.54	8.3	3
High	P08571	0	7.101	7	2	3	2	1	375	40.1	6.23	10.48	2
High	P00734	0	5.619	8	3	3	3	1	622	70	5.9	7.5	3
High	P19823	0	5.467	3	2	3	2	1	946	106.4	6.86	4.14	2
High	P05156	0	5.052	4	2	2	2	1	583	65.7	7.5	5.19	2
High	P06681	0	4.32	3	2	2	2	1	752	83.2	7.42	4.09	2
High	Q9UGM5	0	4.166	5	2	2	2	1	382	42	6.83	4.58	2
High	Q96IY4	0	2.599	4	2	2	2	1	423	48.4	7.71	4.81	2
High	P35527	0	6.3	5	1	1	1	1	623	62	5.24	5.21	1
High	P02753	0	5.961	9	1	1	1	1	201	23	6.07	5.13	1
High	P05452	0	3.797	10	1	1	1	1	202	22.5	5.67	1.95	1
High	P0DOX2	0	3.074	2	1	1	1	1	455	48.9	6.81	2.16	1
High	P05154	0	2.264	3	1	1	1	1	406	45.6	9.26	2.37	1
High	A0A0A0MRZ8	0	2.18	8	1	1	1	1	115	12.6	5.29	2.23	1
High	P18428	0	2.174	4	1	1	1	1	481	53.4	6.7	2.39	1
High	P0DOX6	0	2.046	3	1	1	1	1	576	63.4	7.87	0	1
High	P51884	0	1.866	2	1	1	1	1	338	38.4	6.61	2.49	1
High	A0A0C4DH55	0	1.84	8	1	1	1	1	119	13.1	5.94	2.54	1
Medium	P01700	0.011	1.401	11	1	1	1	1	117	12.3	5.91	2.59	1
Medium	Q14376	0.011	1.393	2	1	1	1	1	348	38.3	6.73	1.82	1
Medium	P00740	0.011	1.282	2	1	1	1	1	461	51.7	5.47	2.44	1
Medium	Q9GZX5	0.021	1.197	2	1	1	1	1	532	60	8.62	0	1
Low	Q13464	0.071	0.75	2	1	1	1	1	1354	158.1	5.9	0	1

Supplemental Table 2. Blood typing test between native RBC and cRBC produced by different systems

Donor	RBC type	Blood Group							
		A	B	RhD	RhE	RhC	Rhe	Rhc	K
Donor 1 (PBMC)	Native RBC	-	+	+	-	+	+	+	-
	cRBC (Serum)	-	+	+	-	+	+	+	-
	cRBC (LDL)	-	+	+	-	+	+	+	-
	cRBC (Cholesterol)	-	+	+	-	+	+	+	-
Donor 2 (PBMC)	Native RBC	-	+	+	+	+	+	+	-
	cRBC (Serum)	-	+	+	+	+	+	+	-
	cRBC (LDL)	-	+	+	+	+	+	+	-
	cRBC (Cholesterol)	-	+	+	+	+	+	+	-
Donor 3 (CBMC)	Native RBC	-	-	+	-	+	+	+	-
	cRBC (Serum)	-	-	+	-	+	+	+	-
	cRBC (LDL)	-	-	+	-	+	+	+	-
	cRBC (Cholesterol)	-	-	+	-	+	+	+	-
